# Transcriptome Analyses of Chicken Primary Macrophages Infected With Attenuated *Salmonella* Typhimurium Mutants

**DOI:** 10.3389/fmicb.2022.857378

**Published:** 2022-05-03

**Authors:** Bijit K. Bhowmik, Arvind Kumar, Dharanesh Gangaiah

**Affiliations:** Discovery Bacteriology and Microbiome, Elanco Animal Health Inc., Greenfield, IN, United States

**Keywords:** *Salmonella* Typhimurium, oral vaccine, RNA-seq, transcriptome, primary macrophage

## Abstract

*Salmonella enterica* is one of the most common foodborne illnesses in the United States and worldwide, with nearly one-third of the cases attributed to contaminated eggs and poultry products. Vaccination has proven to be an effective strategy to reduce *Salmonella* load in poultry. The *Salmonella* Typhimurium Δ*crp*-*cya* (MeganVac1) strain is the most commonly used vaccine in the United States; however, the mechanisms of virulence attenuation and host response to this vaccine strain are poorly understood. Here, we profiled the invasion and intracellular survival phenotypes of Δ*crp*-*cya* and its derivatives (lacking key genes required for intra-macrophage survival) in HD11 macrophages and the transcriptome response in primary chicken macrophages using RNA-seq. Compared to the parent strain UK1, all the mutant strains were highly defective in metabolizing carbon sources related to the TCA cycle and had greater doubling times in macrophage-simulating conditions. Compared to UK1, the majority of the mutants were attenuated for invasion and intra-macrophage survival. Compared to Δ*crp*-*cya*, while derivatives lacking *phoPQ, ompR-envZ, feoABC* and *sifA* were highly attenuated for invasion and intracellular survival within macrophages, derivatives lacking *ssrAB, SPI13, SPI2, mgtRBC, sitABCD, sopF, sseJ* and *sspH2* showed increased ability to invade and survive within macrophages. Transcriptome analyses of macrophages infected with UK1, Δ*crp*-*cya* and its derivatives lacking *phoPQ, sifA* and *sopF* demonstrated that, compared to uninfected macrophages, 138, 148, 153, 155 and 142 genes were differentially expressed in these strains, respectively. Similar changes in gene expression were observed in macrophages infected with these strains; the upregulated genes belonged to innate immune response and host defense and the downregulated genes belonged to various metabolic pathways. Together, these data provide novel insights on the relative phenotypes and early response of macrophages to the vaccine strain and its derivatives. The Δ*crp*-*cya* derivatives could facilitate development of next-generation vaccines with improved safety.

## Introduction

Non-typhoidal Salmonellosis is one of the most common foodborne illnesses worldwide, accounting for an estimated 1.35 million infections, 26,500 hospitalizations, 420 deaths, and 400 million losses from direct medical costs each year in the United States alone (National Center for Emerging and Zoonotic Infectious Diseases (NCEZID), 2019; WHO, 2020). Caused by serovars belonging to *Salmonella enterica* subspecies *enterica*, non-typhoidal Salmonellosis typically presents as self-limiting gastroenteritis with diarrhea, fever and abdominal cramps as the primary symptoms. Although uncommon, invasive infections with life-threatening complications with bacteremia and extra-intestinal manifestations can occur in risk populations such as infants, young children, the elderly and immunocompromised patients (Gordon, 2008). Several serotypes have been associated with Salmonellosis, with *S*. Enteritidis as the most frequent one followed by *S*. Typhimurium (Su and Chui, 2007; Ryan et al., 2017). *Salmonella* has a broad host range and colonizes the gastrointestinal tract of a variety of domestic and wild animals, including poultry, without any detectable symptoms (Barrow et al., 2012). Humans usually contract *Salmonella* by consuming contaminated food of animal origin; poultry meat and eggs are believed to be the primary source of human infections (Antunes et al., 2016).

As an intercellular pathogen with a broad host range, *Salmonella* has evolved to survive in very different and harsh environments. After ingestion, *Salmonella first* encounters the highly acidic gastric juice in the stomach. To circumvent this acidic pH and maintain pH homeostasis, *Salmonella* upregulates several amino acid decarboxylase systems (Park, 1996; Jonge et al., 2003; Kieboom and Abee, 2006; Morita, 2006; Álvarez-Ordóñez et al., 2010) and induces synthesis of acid shock proteins including RpoS and PhoPQ (Audia and Webb, 2001; Tu et al., 2006). Changes in the composition of membrane fatty acids and the resulting changes in membrane fluidity also play a vital role in the survival of *Salmonella* in low pH (Álvarez-Ordóñez et al., 2008; Alonso-Hernando and Alonso-Calleja, 2010). Once *Salmonella* reaches the proximal intestine, it encounters bile which possesses strong antimicrobial properties (Begley and Gahan, 2005; Merritt and Donaldson, 2009). *Salmonella* is inherently resistant to bile due to the upregulation of genes encoding two-component signal transduction systems, efflux pumps, and various transcriptional regulators (Begley and Gahan, 2005). *Salmonella* also upregulates several genes to survive in the high salt and low oxygen environments of the intestine (Frymier et al., 1997; Wei and Miller, 1999; Ševčik et al., 2001; Balaji et al., 2005; Su et al., 2009). In the intestine, *Salmonella* invades intestinal epithelial cells and dendritic cells and induces its own uptake by antigen sampling M cells; of these, uptake via M cells is the preferred route of entry for *Salmonella*. *Salmonella* uses its long polar fimbriae to attach to M cells (Jones and Gori, 1994; Bäumler et al., 1996) and induces membrane ruffles, which engulf the bacteria and result in endocytosis (Jepson and Clark, 1998). In addition, *Salmonella* has the ability to invade intestinal epithelial cells using both the trigger mechanism (Type III secretion system-dependent) and zipper mechanism (Type III secretion system-independent) (McGhie et al., 2009; Rosselin et al., 2011; Moest and Meresse, 2013). *Salmonella* can also modify the intestinal epithelial cells into M cells, thus promoting its own uptake (Tahoun et al., 2012). M cells are located on top of the Payer's patches, and actively transport bacteria to underlying macrophages.

**Figure 1:**
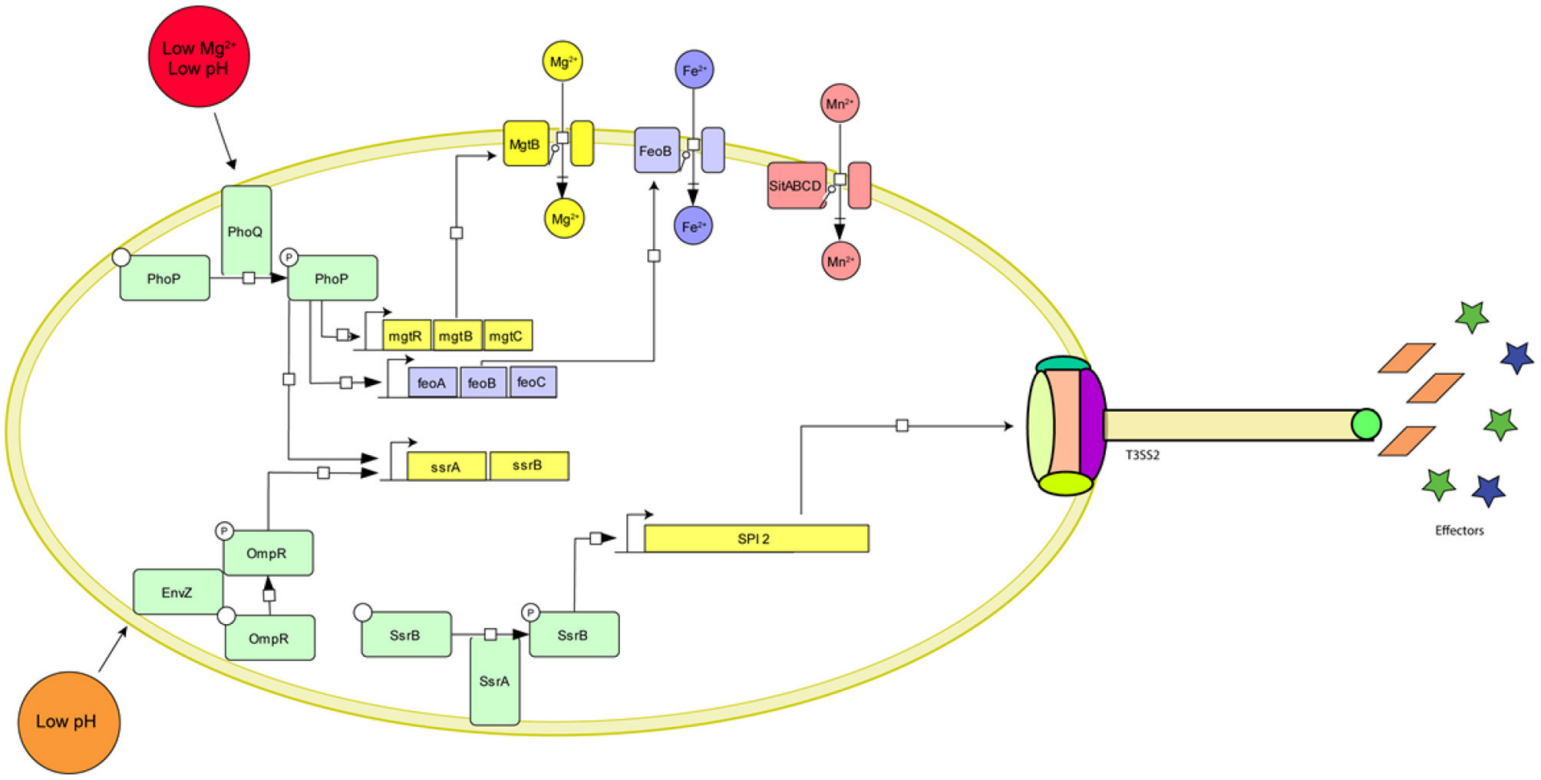
Graphical representation of the major *Salmonella* genes required for survival inside macrophages and their environmental cues. The two master regulators, PhoPQ and OmpR-EnvZ sense the low pH and Mg^2+^ concentration inside the SCV and initiate a cascade of gene activation resulting in the upregulation of ion importers (MgtRBC, FeoABC, and SitABCD), SPI activation (SPI2, SPI13), formation of the Type III secretion system needle complex and expression of effector molecules.

Once *Salmonella* enters the macrophages, it resides inside a modified endosome known as *Salmonella* Containing Vacuole (SCV). Inside this SCV, *Salmonella* alters the expression of a plethora of genes to adapt itself to survive and replicate (Alpuche-Aranda et al., 1994; Meresse et al., 2001; Srikumar et al., 2015). The low magnesium and low iron concentration inside SCV activate the PhoPQ system. This two-component system then upregulates the expression of *mgtRBC* and *feoABC* operons, which lead to increased uptake of Mg^2+^ and Fe^2+^, respectively (Groisman, 2001; Choi et al., 2009). The deficiency of manganese inside the SCV also induces the expression of the ABC type transporter *sitABCD* (David, 2002; Ikeda et al., 2005). The sensor kinase, EnvZ senses the acidic environment of SCV and engages OmpR to upregulate the expression of various pH regulatory genes resulting in acidification of the bacterial cytosol (Chakraborty et al., 2015). OmpR also upregulates the expression of the two-component system SsrAB (Lee et al., 2000). SsrAB in turn acts as a master regulator and upregulates the expression of genes in *Salmonella* Pathogenicity Island 2 (SPI2) (Worley et al., 2000; Walthers et al., 2007). SPI2 encodes for the Type III secretion system that delivers at least 28 effector molecules (Figueira, 2012; Fàbrega and Vila, 2013). These effectors are required for SCV maturation and maintenance (eg. SifA, SopF, SseJ), bacterial growth and replication (eg. SteA), host-cell modification (eg. SpvB, SteC), and inhibiting host immune response (eg. sspH2, SpvC, GtgA, SseK1, SseK2, SseK3, SspH1, GogB, and SpvD), reviewed in (Jennings et al., 2017) and summarized in Figure 1.

The above-described strategies allow *Salmonella* to successfully evade host defense mechanisms and survive within macrophages, contributing to long-term persistence in poultry and consequent transmission to humans. Vaccination has proven to be an effective strategy to reduce *Salmonella* load in poultry. The *S*. Typhimurium *crp*-*cya* mutant (MeganVac1; also referred to here as Δ*crp*-*cya*) is the first and the most commonly used live attenuated vaccine in the United States (Hassan and Curtiss, 1990, 1996; Curtiss et al., 1993; Curtiss and Hassan, 1996; Dorea et al., 2010). The Δ*crp*-*cya* mutant is highly attenuated for infection (safe) and persistence in the host (no carrier status) and is known to elicit both humoral and cell-mediated immunity against strains from both homologous and heterologous serotypes (immunogenic) (Hassan and Curtiss, 1990, 1996; Curtiss et al., 1993; Curtiss and Hassan, 1996; Dorea et al., 2010). Despite being a successful vaccine, relatively little is known about the infection phenotypes and early response of macrophages to this vaccine strain.

Here, we characterized the invasion and intracellular survival phenotypes of the Δ*crp*-*cya* mutant and its derivatives lacking key genes required for intra-macrophage survival using HD11 chicken macrophage cells. We also profiled the transcriptome response of macrophages to Δ*crp*-*cya* and its derivatives Δ*crp*-*cya*-Δ*phoPQ*, Δ*crp*-*cya*-Δ*sifA* and Δ*crp*-*cya*-Δ*sopF* in primary chicken macrophages using RNA-sequencing.

## Materials and Methods

### Bacterial Strains and Culture Conditions

The bacterial strains used in this study are listed in Table 1. *S*. Typhimurium strains were routinely grown overnight in brian heart infusion (BHI) broth supplemented with 0.1% glucose at 37°C with shaking at 200 rpm. *E. coli* strains were grown in Luria-Bertani (LB) broth or BHI broth aerobically at 37°C with shaking at 200 rpm. Where applicable, the media was supplemented with chloramphenicol (25 μg/mL for *S*. Typhimurium and 50 μg/mL for *E. coli*).

**Table 1:** Bacterial strains and plasmids used in this study.

**Strain**	**Genotype**	**Reference or source**
*S*. Typhimurium UK1	Parent strain (ATCC 68169)	ATCC
*S*. Typhimurium UK1 Δ*crp-cya*	*crp::Tn10-cya::Tn10* (MeganVac1)	Elanco Animal Health, Inc.
*S*. Typhimurium UK1 Δ*crp-cya-*Δ*phoPQ*	*crp::Tn10-cya::Tn10-phoPQ*::*CmR*	This study
*S*. Typhimurium UK1 Δ*crp-cya-*Δ*ompR*-*envZ*	*crp::Tn10-cya::Tn10-ompR-envZ::CmR*	This study
*S*. Typhimurium UK1 Δ*crp-cya-*Δ*ssrAB*	*crp::Tn10-cya::Tn10-ssrAB::CmR*	This study
*S*. Typhimurium UK1 Δ*crp-cya-*Δ*SPI13*	*crp::Tn10-cya::Tn10-SPI13::CmR*	This study
*S*. Typhimurium UK1 Δ*crp-cya-*Δ*SPI2*	*crp::Tn10-cya::Tn10-SPI2::CmR*	This study
*S*. Typhimurium UK1 Δ*crp-cya-*Δ*mgtRBC*	*crp::Tn10-cya::Tn10-mgtRBC::CmR*	This study
*S*. Typhimurium UK1 Δ*crp-cya-*Δ*feoAC*	*crp::Tn10-cya::Tn10-feoAC::CmR*	This study
*S*. Typhimurium UK1 Δ*crp-cya-*Δ*sitABCD*	*crp::Tn10-cya::Tn10-sitABCD::CmR*	This study
*S*. Typhimurium UK1 Δ*crp-cya-*Δ*sifA*	*crp::Tn10-cya::Tn10-sifA::CmR*	This study
*S*. Typhimurium UK1 Δ*crp-cya-*Δ*sopF*	*crp::Tn10-cya::Tn10-sopF::CmR*	This study
*S*. Typhimurium UK1 Δ*crp-cya-*Δ*sspH2*	*crp::Tn10-cya::Tn10-sspH2::CmR*	This study
*S*. Typhimurium UK1 Δ*crp-cya-*Δ*sseJ*	*crp::Tn10-cya::Tn10-sseJ::CmR*	This study
*S*. Typhimurium UK1 Δ*crp-cya* (pUC_mCherry)	*crp::Tn10-cya::Tn10* mutant containing pUC_mCherry plasmid	This study
pSIJ8	Plasmid carrying inducible λ red recombinase	Jensen et al., 2015
pUC19	Vector used for general cloning purposes	GenScript, Inc.
pEL01	pUC19 *phoPQ::CmR*	This study
pEL02	pUC19 *ompR-envZ::CmR*	This study
pEL03	pUC19 *ssrAB::CmR*	This study
pEL04	pUC19 *SPI13::CmR*	This study
pEL05	pUC19 *SPI2::CmR*	This study
pEL06	pUC19 *mgtRBC::CmR*	This study
pEL07	pUC19 *feoABC::CmR*	This study
pEL08	pUC19 *sitABCD::CmR*	This study
pEL09	pUC19 *sifA::CmR*	This study
pEL10	pUC19 *sopF::CmR*	This study
pEL11	pUC19 *sspH2::CmR*	This study
pEL12	pUC19 *sseJ::CmR*	This study

### HD11 Cells and Culture Conditions

HD11 cells were procured from the U. S. Department of Agriculture. HD11 is a chicken macrophage-like cell line that was derived from chicken hematopoietic cells after *in vitro* transformation with the avian myelocytomatosis type MC29 virus (Beug et al., 1979). HD11 cells were maintained in the Iscove Modified Dulbecco Media (IMDM, Gibco) medium supplemented with 10% fetal bovine serum (FBS) at 39°C in a humidified 5% CO_2_ incubator.

### Construction of Δ*crp*-*cya* Derivatives

**Figure 2:**
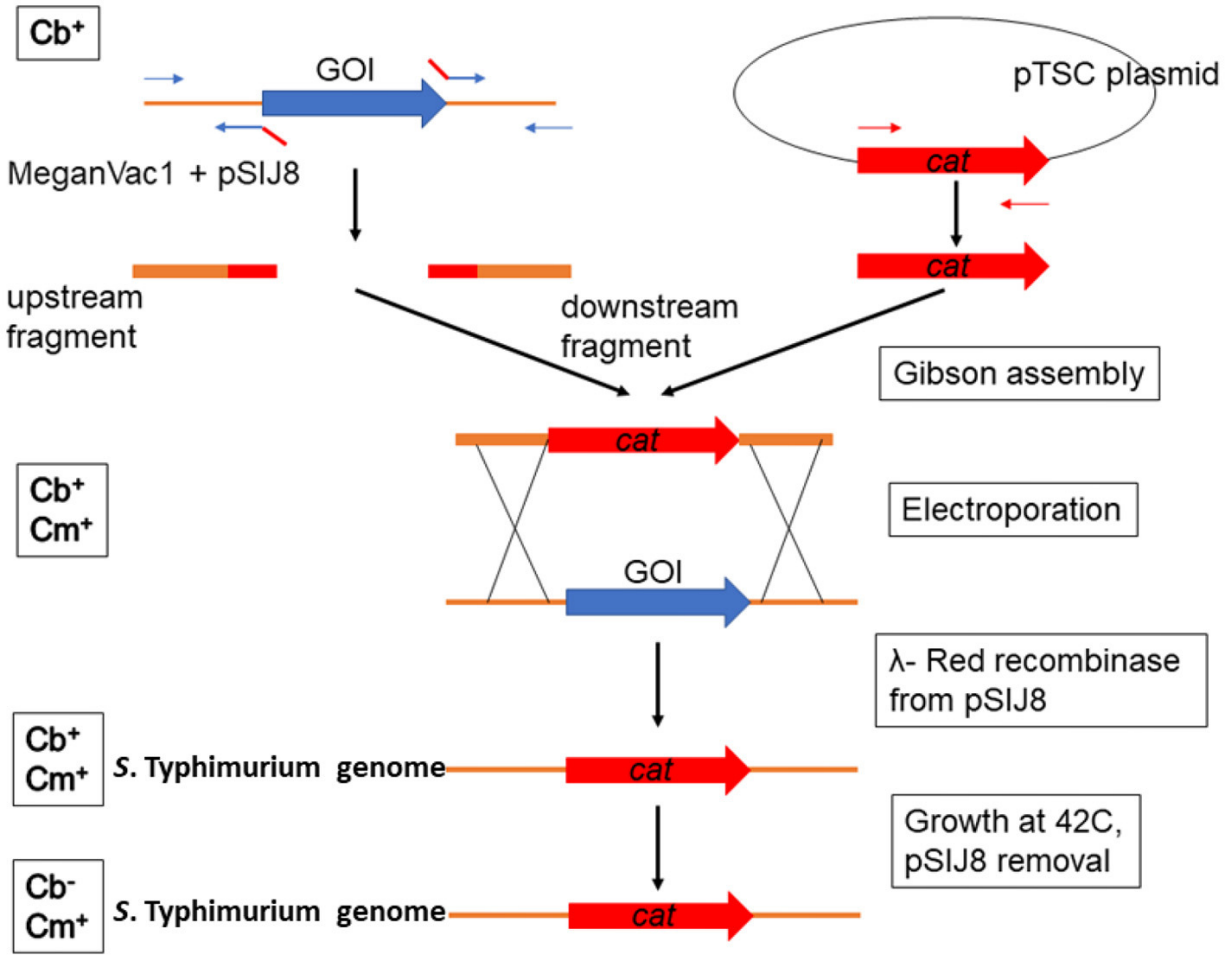
Schematic diagram showing the construction of *S*. Typhimurium UK1 Δ*crp*-*cya* derivatives. λ-red recombinase mediated homologous recombination was used to replace the gene of interest (GOI) with a chloramphenicol cassette. Abbreviations in the left side boxes suggest sensitivity toward carbenicillin (Cb) and chloramphenicol (Cm).

All the plasmids and primers used in this study are described in Table 1 and Supplementary Table 1, respectively. The commercial vaccine *S*. Typhimurium Δ*crp*-*cya* mutant (MeganVac1) was used as the parent strain for all the deletions reported in this study (Curtiss and Kelly, 1987). The Δ*crp*-*cya* mutant was constructed from the pathogenic *S*. Typhimurium strain UK-1 (Luo et al., 2011). For this study, the UK-1 strain was purchased from ATCC (ATCC 68169) and used as a control. To construct *S*. Typhimurium mutants, the λ-red recombinase-mediated recombination was used (Figure 2) (Datsenko and Wanner, 2000). In short, approximately 500-bp upstream and 500-bp downstream regions of the gene of interest were first amplified using PCR using the Δ*crp*-*cya* mutant genomic DNA as a template. Chloramphenicol expressing gene (*cat*) was amplified from the pTSC plasmid (Yan et al., 2008). These DNA fragments were assembled with the pUC19 backbone (NEB) using a commercial Gibson Assembly kit (NEB), where the upstream and the downstream regions flanked the chloramphenicol resistant gene. The whole cassette was then amplified using PCR and electroporated into the Δ*crp*-*cya* mutant cells harboring the λ-red recombinase encoding plasmid pSIJ8 (Jensen et al., 2015). The expression of λ-red recombinase was induced by adding 50 mM of arabinose. Throughout the process, the Δ*crp*-*cya* mutant cells were maintained at 30°C. After 2 h of incubation at 30°C, the transformants were plated on BHI-agar plates supplemented with 50μg/mL of chloramphenicol. Successful transformants were verified using PCR followed by Sanger sequencing. The pSIJ8 plasmid was then cured by sub-culturing the mutants at 42°C overnight for five passages. Depletion of the plasmid was verified by carbenicillin sensitivity test (pSIJ8 has a functional *bla* gene) followed by lack of PCR amplification of the plasmid backbone.

### Growth Kinetics Analyses

The *S*. Typhimurium mutants were grown overnight in BHI medium supplemented with 0.1% glucose at 37°C while shaking. The following day, cells were washed twice with Dulbecco′s Phosphate Buffered Saline (DPBS, Gibco) and resuspended in M9 minimal medium (M9MM) supplemented with 1% glucose and 0.12% casamino acids. Approximately 20,000 bacteria in 200 μL of medium were added into each well of a 96-well plate and the OD_600_ values were measured every hour for 24 h at 30°C using a SpectraMax^®^ i3x multi-plate reader (Molecular Devices). To simulate the SCV environment, growth kinetics of UK-1, the Δ*crp cya* mutant and its derivative mutants were determined using a defined “Phosphate Carbon Nitrogen” medium (PCN) as described previously (Löber et al., 2006). The PCN media contained 80 mM morpholineethanesulfonic acid (MES), pH 4.8, 4 mM Tricine, 100 μM FeCl_3_, 376 μM K_2_SO_4_, 50 mM NaCl, 0.4 mM K_2_HPO_4_/KH_2_PO_4_, pH 4.8, 0.4% glucose, 15 mM NH_4_Cl, 1 mM MgSO_4_ and 10 μM CaCl_2_. A cocktail of micronutrients was also added to the media. The composition of micronutrients was 10 nM Na_2_MoO_4_.2H_2_O, 10 nM NaSeO_3_, 4 nM H_3_BO_3_, 300 nM CoCl_2_.6H_2_O, 100 nM CuSO_4_.5H_2_O, 800 nM MnCl_2_ and 1nM ZnSO_4_. Final pH was adjusted to 5.8. The growth kinetics of all the *Salmonella* mutants was monitored using SpectraMax® i3x multi-plate reader as described above.

### Carbon Source Utilization Assays

The ability of the *S*. Typhimurium mutants to metabolize different carbon sources was investigated using PM1 and PM2A MicroPlate™ (Biolog Inc.). The plates were set up according to the manufacturer's protocol. In short, the bacterial cells were grown overnight on BHI-agar plates. Single colonies were then streaked on BHI-agar plates covering the whole surface. The culture was then scraped with a sterile cotton swab and resuspended in 5 mL of inoculation fluid (IF0) until turbidity (T) of 42% was reached. Three ml of 42% T cell suspension was then mixed with 1 mL IF0 + dye. One hundred microliters of cell suspension was then added into each well of the PM1 and PM2A plates. Plates were then incubated at 37°C overnight. Cell growth was analyzed by visual inspection of color change.

### Invasion and Intracellular Survival Assays

The HD11 chicken macrophage cells were maintained in IMDM (Gibco) medium supplemented with 10% FBS (Gibco). The day before infection, approximately 1 × 10^5^ cells were seeded in each well of a 24-well plate and incubated at 39°C under 5% CO_2_ atmosphere. HD11 cells were activated by adding phorbol 12-myristate 13-acetate (PMA, Sigma) at a concentration of 100 ng/mL (Wisner et al., 2011). Overnight grown *S*. Typhimurium mutants were sub-cultured and grown until they reached an OD_600_ of 1.0. Bacterial cells were then washed twice with DPBS and resuspended in IMDM medium supplemented with 10% heat-inactivated FBS. HD11 cells were also washed twice with DPBS and the bacteria were added to each well at a multiplicity of infection (MOI) of 10. To increase contact between the macrophages and the bacteria, the plates were centrifuged at 600 × *g* for 5 min. The plates were then incubated at 39°C for 30 min. After infection, the extracellular bacteria were removed by washing twice with DPBS and replacing the medium with fresh complete IMDM medium supplemented with 100 μg/mL of gentamicin. To determine invasion of *S*. Typhimurium mutants, after incubation for an hour, the medium was removed, cells were washed twice with DPBS, 1 mL of DPBS containing 1% of Triton X-100 was added and incubated for 5 min at room temperature, the lysate was serially diluted, plated on BHI-agar plates and incubated at 37°C under aerobic conditions. CFU counts were quantified for each mutant and the results were expressed as fold change in recovered CFUs compared to UK1 strain. To determine *S*. Typhimurium survival and replication inside macrophages, after incubation for an hour with gentamicin, the medium was again removed and replaced with a fresh complete IMDM medium containing 30 μg/mL gentamicin and incubated for 24 h. CFUs were quantified from HD11 cells infected with *S*. Typhimurium mutants 24 h post-infection as described above.

### Isolation of Chicken Peripheral Blood Mononuclear Cells (PBMCs) and Their Differentiation Into Macrophages

Chicken PBMCs were isolated and cultured according to a previous protocol (Wigley et al., 2002; Feng et al., 2017; Peng et al., 2020). Briefly, blood was collected from healthy chickens and heparinized blood was mixed with an equal volume of DPBS. Ten ml of this suspension was then carefully layered on top of 10 mL Histopaque®-1077 (Sigma) in a 50 mL conical tube. Tubes were then centrifuged at 400 × *g* for 30 min at room temperature with the lowest acceleration and no braking. After centrifugation, the top plasma layer was removed and the interface was collected in a fresh tube. The collected lymphocytes were then mixed gently with 5 volumes of DPBS and centrifuged at 250 × *g* for 10 min at room temperature. The supernatant was discarded, and the pellet was washed twice with DPBS at room temperature. The pellet was then finally resuspended in RPMI 1640 Medium, supplemented with GlutaMAX™ (ThermoFisher Sc.), 10% FBS (Gibco), Anti-anti (ThermoFisher Sc.) and 50 μg/mL chicken granulocyte-macrophage colony-stimulating factor (GMCSF, Abcam). The viability of the cells was determined by Trypan Blue exclusion and approximately 1 × 10^7^ cells were transferred to a 24 well plate and incubated at 41°C with 5% CO_2_. The spent medium was replaced with a fresh medium supplemented with GMCSF every 2 days and the morphology of the adherent cells was monitored using an inverted microscope. After 7 days of incubation, the cells were dislodged from the surface and analyzed using flow cytometry. Macrophages were identified by staining the cells with chicken macrophage marker KUL01 (ThermoFisher Scientific) and analyzing using a flow cytometer (BD FACSAria) as described previously (Mast et al., 1998).

### Transcriptome Profiling

Transcriptome analyses was performed using seven-day old, macrophages differentiated as described above (Huang et al., 2019). Approximately 5 × 10^4^ macrophages were seeded in a 24-well plate and incubated overnight at 41°C with 5% CO_2_. Overnight grown *S*. Typhimurium cells were concentrated and washed twice with DPBS. Approximately 1 × 10^6^ bacteria were then resuspended in 1 mL of 10% chicken serum in DPBS and incubated for 30 min at 37°C. Bacterial cells were then washed twice in DPBS and resuspended in 1 mL RPMI 1,640 medium with 10% heat-inactivated FBS. The primary macrophages were infected with the *S*. Typhimurium mutants with an MOI of 10 for 30 min. After 30 min, extracellular bacterial were killed by adding 100 μg/ml of gentamicin for an hour and the medium was then replaced with fresh RPMI-1640 medium supplemented with 30 μg/mL gentamicin and incubated for another hour. Cells were then washed twice with DPBS, lysed by using the RLT buffer (Qiagen) and shipped on dry ice to Cofactor Genomics for RNA-seq (http://cofactorgenomics.com, St. Louis, MO). To confirm the uptake of *Salmonella* by primary macrophages, in parallel, we also infected macrophages with the Δ*crp*-*cya* mutant cells constitutively expressing mCherry with an MOI of 10 and monitored under a fluorescent microscope 2 hours post-infection.

The samples were processed by Cofactor Genomics as described previously (do Amaral et al., 2020). Briefly, total RNA was isolated using Qiagen RNAeasy Mini Kit (Qiagen) following the manufacturer's instructions. The isolated RNA was then treated with DNase I (Thermo Fisher Scientific) to remove potential DNA contamination. RNA was then precipitated using lithium chloride (Sigma). The concentration and quality of RNA were determined using nanophotometer.

RNA samples with RNA integrity number (RIN) > 8.0, A_260_/A_280_ > 1.9 and A_260_/A_230_ > 2 were selected for mRNA library preparation and Next Generation Sequencing. Briefly, the total RNA was incubated with mRNA capture beads to remove contaminating ribosomal RNA from the sample using the Kapa Stranded mRNA-Seq kit (Kapa Biosystems) following manufacturer's instructions. The resulting poly(A)-captured mRNA was then fragmented. First-strand cDNA synthesis was performed using reverse transcriptase and random primers in the presence of Actinomycin D, followed by second-strand cDNA synthesis with DNA polymerase I and RNase H. Double-stranded cDNA was end-repaired and A-tailed for subsequent adaptor ligation. Indexed adaptors were ligated to the A-tailed cDNA. Enrichment by PCR was performed to generate the final cDNA sequencing library. Libraries were sequenced as paired-end 150 base reads on an Illumina NextSeq500 following the manufacturer's protocols.

Reads were aligned, quantified and analyzed using CLC Genomics Workbench (Qiagen). Differential gene expression among samples was determined using the DESeq2 package for R in Rstudio (v. 1.0.136) (Love et al., 2014). Data were normalized with DESeq2 algorithms. Transcripts with less than one raw count were excluded, and the default DESeq2 algorithms were used to remove outlier transcripts based on Cook's distances. The Benjamini and Hochberg algorithm was used to control the false discovery rate (FDR) and transcripts were considered differentially expressed if the expression between two treatments differed by at least 2-fold with FDR < 0.1 (Witten et al., 2012). NetworkAnalyst program was used to perform pathway enrichment of differentially expressed genes (Xia et al., 2014).

The data from the RNA-seq experiments were deposited at the NCBI Gene Expression Omnibus (GEO) database under the series record GSE193063 and accession numbers GSM5772479 to GSM5772490.

## Results

### Construction of Δ*crp*-*cya* Derivatives

**Figure 3:**
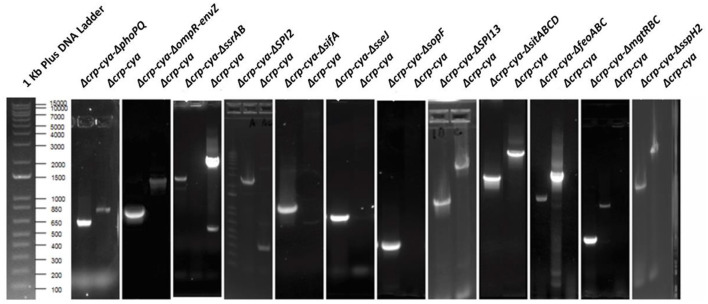
Agarose gel electrophoresis images of confirmation of gene deletions in *S*. Typhimurium UK1 Δ*crp*-*cya* derivatives. PCR amplification was performed to confirm the deletions. For Δ*crp*-*cya*-Δ*phoPQ*, Δ*crp*-*cya*-Δ*ompR-envZ*, Δ*crp*-*cya*-Δ*ssrAB*, Δ*crp*-*cya*-Δ*SPI13*, Δ*crp*-*cya*-Δ*sitABCD*, Δ*crp*-*cya*-Δ*feoABC*, Δ*crp*-*cya*-Δ*mgtRBC* and Δ*crp*-*cya*-Δ*sspH2*, the primers were designed flanking the genes of interest. For Δ*crp*-*cya*-ΔSPI2, Δ*crp*-*cya*-Δ*sifA*, Δ*crp*-*cya*-Δ*sopF*, and Δ*crp*-*cya*-Δ*sseJ*, the forward primer was designed to bind to *cat* gene and the reverse primer was designed to bind to the 3' region of the target gene on the chromosome.

The Δ*crp*-*cya* mutant is one of the most commonly used and successful live attenuated vaccines used in the United States to reduce *Salmonella* load in poultry. *Salmonella* has evolved several strategies to successfully evade host defense mechanisms within macrophages. Previous studies have identified several key *Salmonella* factors required for survival within macrophages. To better understand the infection phenotypes and early response of macrophages to the Δ*crp*-*cya* mutant and its derivatives lacking key factors required for intra-macrophage survival, we generated *S*. Typhimurium insertional inactivation mutants of *phoPQ* (master regulator), *ompR-envZ* (another master regulator), *feoABC* (iron transporter), *sifA* (SPI2 effector), *ssrAB* (SPI2 regulator), *SPI13* (*Salmonella* pathogenicity island 13), *SPI2* (*Salmonella* pathogenicity island 2), *mgtRBC* (magnesium transporter), *sitABCD* (manganese transporter), *sopF* (T3SS1 effector), *sseJ* (SPI2 effector) and *sspH2* (SPI2 effector) using Δ*crp*-*cya* as the parent strain (Table 1, Figure 1). PCR and sequencing confirmed that all the mutant strains were insertionally inactivated by chloramphenicol cassette and this insertion was in-frame (Figure 3).

### Growth Kinetics of Δ*crp*-*cya* and Its Derivatives

**Figure 4:**
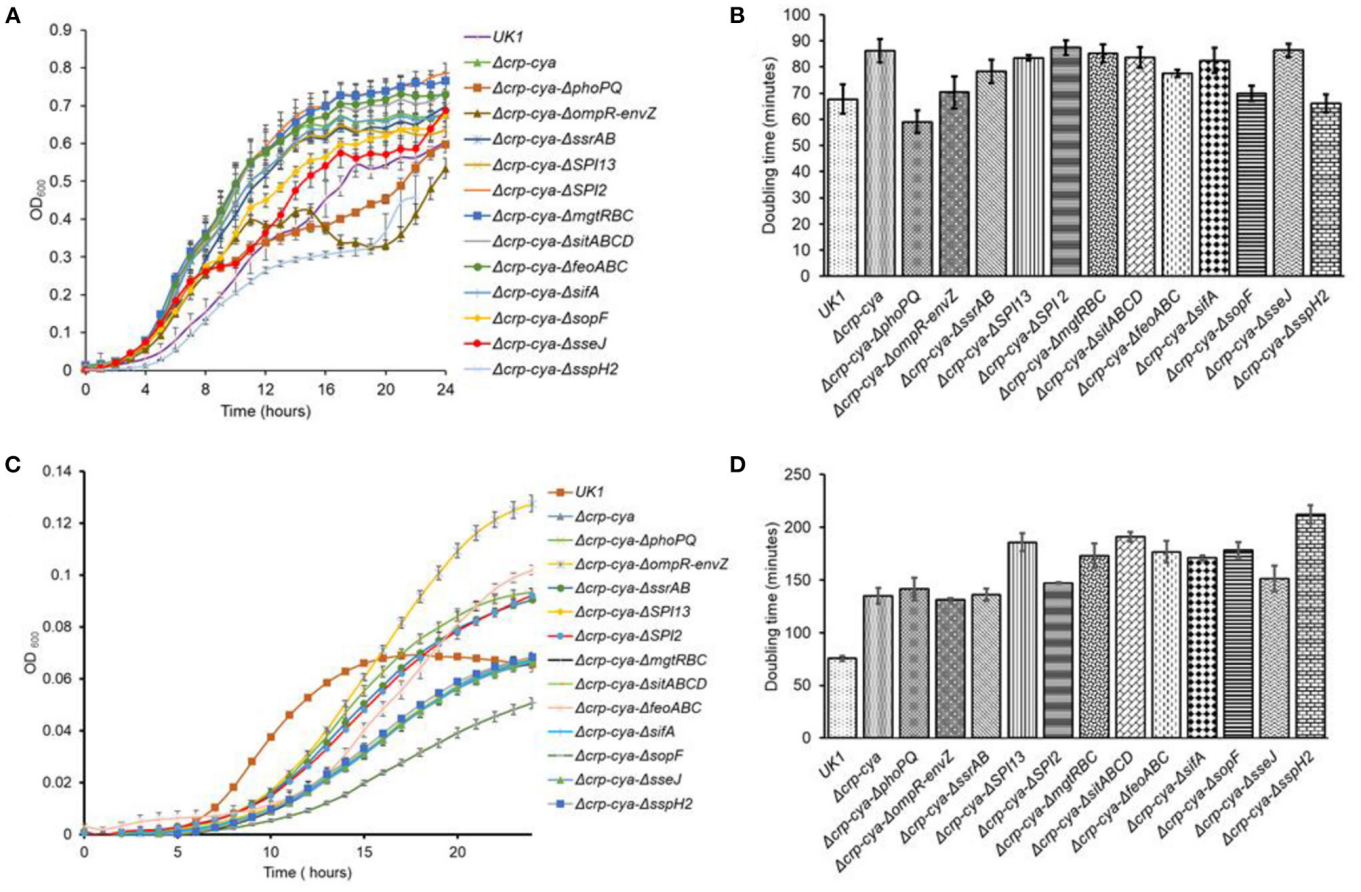
*In vitro* growth characteristics of *S*. Typhimurium UK1, Δ*crp*-*cya* and its derivatives in defined and SCV-simulating media. (**A,B)**. Growth curves and doubling times of *S*. Typhimurium UK1, Δ*crp*-*cya* and its derivatives in M9 medium supplemented with 0.1% glucose and 0.12% casamino acids. **(C)** and **(D)**. Growth curves and doubling times of *S*. Typhimurium UK1, Δ*crp*-*cya* and its derivatives in PCN media.

The *S*. Typhimurium mutants were first tested for their fitness *in vitro* by growing in a defined media (M9MM + glucose). In this media, compared to the parent strain UK1, the majority of the mutants grew well; however, they reached different stationary phase OD_600_ values and presented different doubling times (Figures 4A,B). Interestingly, when the *S*. Typhimurium strains were grown under SCV-simulating conditions, compared to UK1 strain, all the mutants grew poorly, reached significantly different stationary phase OD_600_ values and had significantly greater doubling times (Figures 4C,D).

### Carbon Source Utilization of Δ*crp-cya* and Its Derivatives

The ability of *Salmonella* mutants to utilize different carbon sources is summarized in Table 2. Compared to UK1, the Δ*crp*-*cya* mutant and its derivatives presented significant defects in carbon utilization. Specifically, the Δ*crp*-*cya* mutant and its derivatives were unable to metabolize TCA cycle (succinic acid, malic acid, α- ketoglutaric acid, citric acid and fumaric acid), pentose phosphate pathway (D-glucuronic acid, D-xylose, D-ribose, lactulose, sucrose and α-hydroxy glutaric acid-γ lactone) and glyoxylate pathway (glyoxylic acid) substrates (Table 2). The Δ*crp*-*cya* mutant and its derivatives were also unable to metabolize amino acids that convert into TCA cycle intermediates (L-aspartic acid, L-proline, L-glutamic acid, L-asparagine, L-glutamine, L- histidine and L- alanine) and carbon sources that lead to TCA cycle intermediates (acetic acid, propionic acid, glycolic acid, glyoxylic acid and glycyl-L glutamic acid) (Table 2). Interestingly, while the Δ*crp*-*cya* mutant metabolized glycerol and glycerol 3-phosphate, none of the Δ*crp*-*cya* mutant derivatives metabolized these carbon sources (Table 2).

**Table 2:** Carbon substrate utilization of *Salmonella* Typhimurium, the Δ*crp*-*cya* mutant and its derivatives.

	**UK1**	**Δ*crp-cya***	**Δ*crp-cya-ΔphoPQ***	**Δ*crp-cya-ΔompR-envZ***	**Δ*crp-cya-ΔssrAB***	**Δ*crp-cya-ΔSPI13***	**Δ*crp-cya-ΔSPI2***	**Δ*crp-cya-ΔmgtRBC***	**Δ*crp-cya-ΔsitABCD***	**Δ*crp-cya-ΔfeoABC***	**Δ*crp-cya-ΔsifA***	**Δ*crp-cya-ΔsopF***	**Δ*crp-cya-ΔsseJ***	**Δ*crp-cya-ΔsspH2***
L-arabinose	+	+	+	+	+	+	+	+	+	+	+	+	+	+
N-Acetyl-DGlucosamine	+	+	+	+	+	+	+	+	+	+	+	+	+	+
D-Saccharic acid	+	+	+	+	+	+	+	+	+	+	+	+	+	+
Succinic acid	+													
D-Galactose	+	+	+	+	+	+	+	+	+	+	+	+	+	+
L-Aspartic acid	+													
L-Proline	+													
D-Alanine	+													
D-Trehalose	+	+	+	+	+	+	+	+	+	+	+	+	+	+
D-Mannose	+	+	+	+	+	+	+	+	+	+	+	+	+	+
Dulcitol	+													
D-Serine	+	+	+	+	+	+	+	+	+	+	+	+	+	+
D-Sorbitol	+													
Glycerol	+	+												
L-Fucose	+													
D-Glucuronic acid	+													
D-Gluconic acid	+	+	+	+	+	+	+	+	+	+	+	+	+	+
D,L-α-GlycerolPhosphate	+	+												
D-Xylose	+													
L-Lactic acid	+		+	+	+	+	+	+	+	+	+	+	+	+
Formic acid	+			+	+	+	+	+	+	+	+	+	+	+
D-Mannitol	+	+	+	+	+	+	+	+	+	+	+	+	+	+
L-Glutamic acid	+													
D-Glucose-6- Phosphate	+	+	+	+	+	+	+	+	+	+	+	+	+	+
D-Galactonic Acid-γ-Lactone	+	+	+	+	+	+	+	+	+	+	+	+	+	+
D,L-Malic acid	+													
D-Ribose	+											+		
Tween 20	+													
L-Rhamnose	+													
D-Fructose	+	+	+	+	+	+	+	+	+	+	+	+	+	+
Acetic Acid	+													
α-D-Glucose	+	+	+	+	+	+	+	+	+	+	+	+	+	+
Maltose	+													
D-Melibiose	+													
Thymidine	+	+	+	+	+	+	+	+	+	+	+	+	+	+
L-Asparagine	+													
D-Aspartic acid	+													
D-Glucosaminic acid	+	+	+	+	+	+	+	+	+	+	+	+	+	+
1,2-Propanediol	+													
Tween 40	+													
α-Keto-Glutaric acid	+													
α-Keto-Butyric acid	+													
α-Methyl-DGalactoside	+													
α-D-Lactose	+													
Lactulose	+													
Sucrose	+													
Uridine	+	+	+	+	+	+	+	+	+	+	+	+	+	+
L-Glutamine	+													
m-Tartaric acid	+													
D-Glucose-1- Phosphate	+	+	+	+	+	+	+	+	+	+	+	+	+	+
D-Fructose-6- Phosphate	+	+	+	+	+	+	+	+	+	+	+	+	+	+
Tween 80	+													
α-Hydroxy Glutaric Acid-γLactone	+													
α-Hydroxy Butyric acid	+		+	+	+	+	+	+	+	+	+	+	+	+
ß-Methyl-DGlucoside	+		+	+	+	+	+	+	+	+	+	+	+	+
Adonitol	+													
Maltotriose	+	+		+	+	+		+	+	+	+	+	+	
2-Deoxy Adenosine	+	+	+	+	+	+	+	+	+	+	+	+	+	+
Adenosine	+	+	+	+	+	+	+	+	+	+	+	+	+	+
Glycyl-L-Aspartic acid	+													
Citric acid	+													
myo-Inositol	+													
D-Threonine	+													
Fumaric acid	+													
Bromo succinic acid	+													
Propionic acid	+													
Mucic acid	+													
Glycolic acid	+													
Glyoxylic acid	+													
D-Cellobiose	+													
Inosine	+	+	+	+	+	+	+	+	+	+	+	+	+	+
Glycyl-LGlutamic acid	+				+	+	+	+	+		+	+	+	+
Tricarballylic acid	+													
L-Serine	+	+												
L-Threonine	+													
L-Alanine	+													
L-Alanyl-Glycine	+				+	+	+	+	+		+	+	+	+
Acetoacetic acid	+													
N-Acetyl-ß-DMannosamine	+	+	+	+	+	+	+	+	+	+	+	+	+	+
Mono methyl succinate	+													
Methyl pyruvate	+	+	+	+	+	+	+	+	+	+	+	+	+	+
D-Malic acid	+													
L-Malic acid	+													
Glycyl-L-Proline	+				+	+	+	+	+		+	+	+	+
p-Hydroxy phenyl acetic acid	+													
m-Hydroxy phenyl acetic acid	+													
Tyramine	+													
D-Psicose	+													
L-Lyxose	+													
Pyruvic acid	+	+	+	+	+	+	+	+	+	+	+	+	+	+
Dextrin	+													
Laminarin	+													
Pectin	+													
N-AcetylNeuraminic acid	+													
2-Deoxy-DRibose	+													
3-0-ß-DGalactopyranosylD-arabinose	+			+	+	+	+	+	+	+	+	+	+	+
D-Glucosamine	+	+	+	+	+	+	+	+	+	+	+	+	+	+
Melibionic acid	+													
D-Tartaric acid	+													
L-Tartaric acid	+													
L-Histidine	+													
Hydroxy-LProline	+													

*+ indicates the ability of S. Typhimurium strains to utilize carbon source*.

### Invasion and Intra-Macrophage Survival of Δ*crp-cya* and Its Derivatives

**Figure 5:**
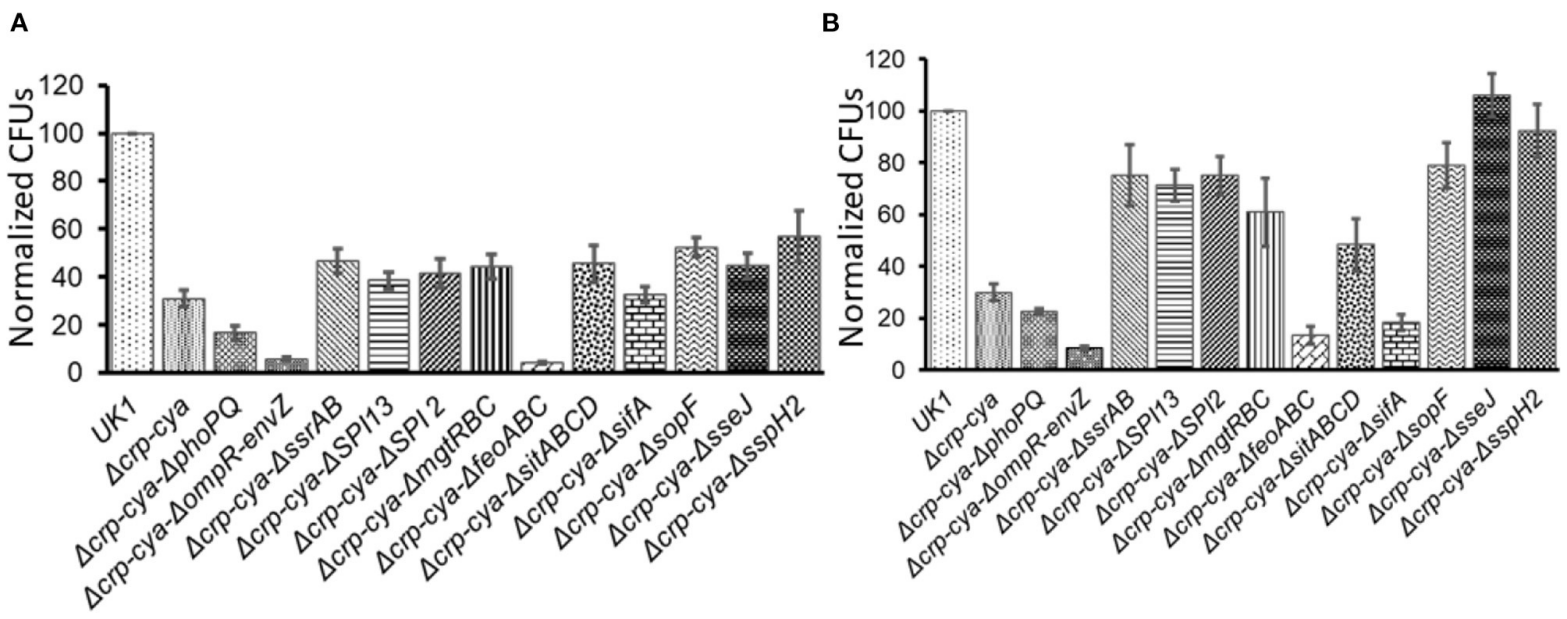
Invasion **(A)** and intra-macrophage survival **(B)** of *S*. Typhimurium UK1, the Δ*crp*-*cya* mutant and its derivatives in HD11 macrophage cells. Normalized CFUs of recovered *Salmonella* strains after 2 h **(A)** and 24 h **(B)** post-infection. Error bars represent the mean ± SD (standard deviation) of three replicates.

To understand the infection phenotypes of the *crp*-*cya* mutant and its derivatives, we assessed the ability of these mutants to invade and survive within macrophages using activated HD11 chicken macrophages. Compared to UK1, all the tested mutants were compromised in their ability to invade macrophages (Figure 5A). Compared to Δ*crp*-*cya* mutant, while Δ*crp*-*cya*-Δ*sifA* showed similar ability to invade macrophages, Δ*crp*-*cya-*Δ*phoPQ*, Δ*crp*-*cya*-Δ*ompR-envZ* and Δ*crp*-*cya*-Δ*feoABC* showed significant reduction in their ability to invade macrophages (Figure 5A). Interestingly, compared to Δ*crp*-*cya* mutant, Δ*crp*-*cya*-Δ*ssrAB*, Δ*crp*-*cya*-Δ*SPI2*, Δ*crp*-*cya*-Δ*SPI13*, Δ*crp*-*cya*-Δ*mgtRBC*, Δ*crp*-*cya*-Δ*sopF*, Δ*crp*-*cya*-Δ*sitABCD*, Δ*crp*-*cya*-Δ*sseJ*, and Δ*crp*-*cya*-Δ*sspH2* displayed increased ability to invade macrophages (Figure 5A).

We also assessed the ability of the Δ*crp*-*cya* mutant and its derivatives to survive within macrophages. Compared to UK1 strain, all the tested mutants, except for Δ*crp*-*cya*-Δ*sseJ*, and Δ*crp*-*cya*-Δ*sspH2*, were compromised in their ability to survive inside macrophages (Figure 5B). Compared to Δ*crp*-*cya* mutant, Δ*crp*-*cya-*Δ*phoPQ*, Δ*crp*-*cya*-Δ*ompR-envZ*, Δ*crp*-*cya*-Δ*sifA* and Δ*crp*-*cya*-Δ*feoABC* showed significant reduction in their ability to survive within macrophages (Figure 5B). Interestingly, compared to Δ*crp*-*cya* mutant, Δ*crp*-*cya*-Δ*ssrAB*, Δ*crp*-*cya*-Δ*SPI2*, Δ*crp*-*cya*-Δ*SPI13*, Δ*crp*-*cya*-Δ*mgtRBC*, Δ*crp*-*cya*-Δ*sopF*, Δ*crp*-*cya*-Δ*sitABCD*, Δ*crp*-*cya*-Δ*sseJ*, and Δ*crp*-*cya*-Δ*sspH2* displayed increased ability to survive inside macrophages (Figure 5B).

### Differentiation of PBMCs Into Macrophages

**Figure 6:**
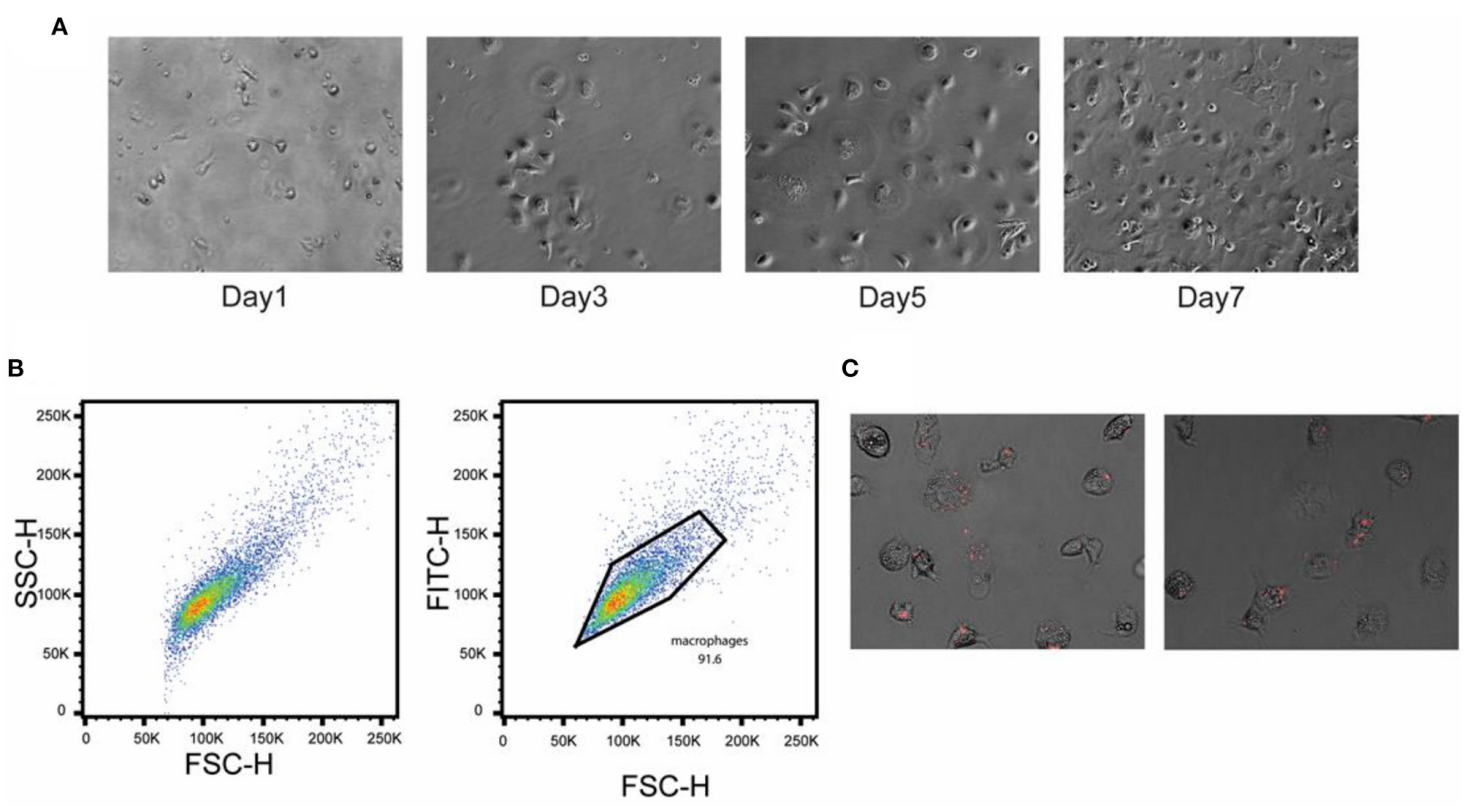
Confirmation of differentiation of chicken PBMCs into primary macrophages. PBMCs were isolated from the heparinized blood collected from healthy birds and separated using Histopaque 1077. **(A)** Phase-contrast images of cultured chicken PBMCs at different time points. **(B)** Flow cytometric analysis showing the pure population of chicken macrophages. Cells were stained with chicken macrophage-specific antibody KUL01. **(C)** Infection of chicken primary macrophages with the Δ*crp*-*cya* mutant cells carrying a mCherry expression plasmid.

To better understand the early response of macrophages to the *crp*-*cya* mutant and its derivatives, PBMCs were isolated from healthy chickens and differentiated into macrophages. On day 1 after seeding, the PBMCs were attached to the bottom of the well (Figure 6A). After 3 days of seeding, the adherent cells became flat and demonstrated classical mammalian M1 macrophage-like morphology (Peng et al., 2020) (Figure 6A). After 5 days, the majority of cells were differentiated into macrophages (Figure 6A). The flow cytometry data indicated that a pure population of macrophages was obtained (Figure 6B). The FSC/SSC analysis demonstrated that the cells were significantly larger than the PBMCs and all the cells were positive for the chicken macrophage marker KUL01 (Figure 6B).

To confirm *Salmonella* uptake and SCV formation, the Δ*crp*-*cya* mutant expressing mCherry was infected and monitored under an inverted microscope. The *Salmonella* were taken up quickly, which subsequently resulted in the formation of numerous small vacuole-like structures inside the primary macrophages, which contained the phagocytosed *Salmonella* (Figure 6C).

### RNA-Seq Analyses of Primary Macrophages Infected With Δ*crp-cya* and Its Derivatives

To better understand the early response of macrophages to Δ*crp-cya* and its derivatives, we profiled the transcriptome of primary macrophages infected with UK1, the Δ*crp*-*cya* mutant and its derivatives lacking *phoPQ, sifA* and *sopF* using RNA-seq. An average of 131 (97–155) million reads were generated for each biological replicate. Approximately, 80–86% of the reads mapped to the reference genome. Of these reads, 69.8–74.2% of the reads mapped to exonic regions, 14.2–18.8% mapped to intronic regions and 11.5–12.6% mapped to intergenic regions. The coefficients of determination (*R*^2^) between replicates and samples ranged from 0.98 to 0.99.

To identify genes differentially expressed in macrophages infected with UK1, Δ*crp*-*cya* Δ*crp*-*cya*-Δ*phoPQ*, Δ*crp*-*cya*-Δ*sifA*, and Δ*crp*-*cya*-Δ*sopF*, we calculated the fold change in expression of genes in macrophages infected with these mutants compared to uninfected macrophages. As detailed in the Materials and Methods section, we used an FDR < 0.1 and a 2-fold change as the cut-off for differential expression.

**Figure 7:**
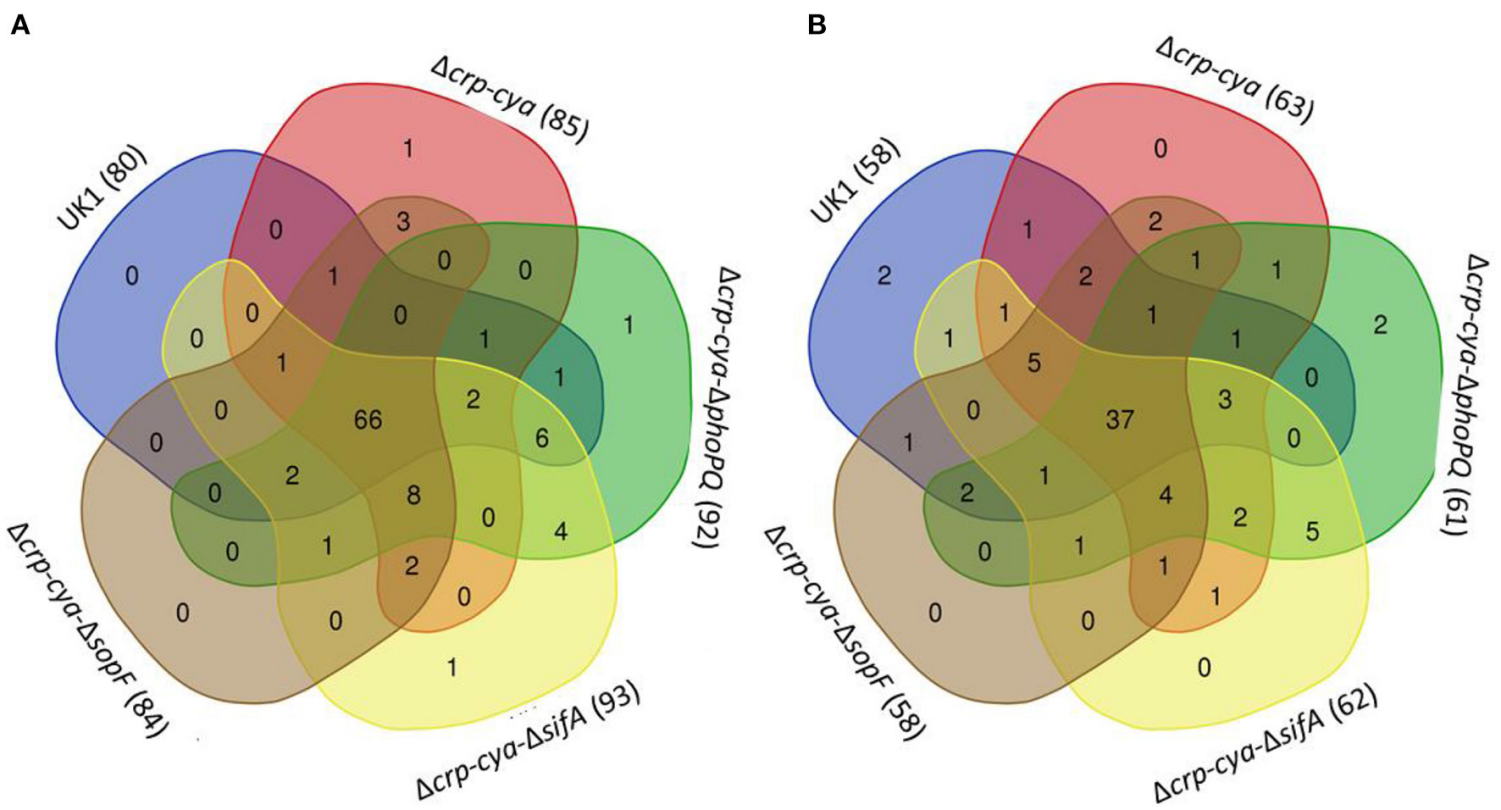
Comparison of the gene expression in *S*. Typhimurium UK1, Δ*crp*-Δ*cya* and its derivatives. **(A)** Venn diagram showing the overlap of genes upregulated in macrophages infected with UK1, Δ*crp*-*cya*, Δ*crp*-*cya*-Δ*phoPQ*, Δ*crp*-*cya*-Δ*sifA* and Δ*crp*-*cya*-Δ*sopF*. The numbers in the bracket next to the strain name indicate the total number of genes upregulated. **(B)** Venn diagram showing the overlap of genes downregulated in macrophages infected with UK1, Δ*crp*-*cya*, Δ*crp*-*cya*-Δ*phoPQ*, Δ*crp*-*cya*-Δ*sifA* and Δ*crp*-*cya*-Δ*sopF*. The numbers in the bracket next to the strain name indicate the total number of genes downregulated.

Comparison of macrophages infected with UK1, Δ*crp*-*cya*, Δ*crp*-*cya*-Δ*phoPQ*, Δ*crp*-*cya*-Δ*sifA* and Δ*crp*-*cya*-Δ*sopF* strains to uninfected macrophages identified 138, 148, 153, 155, and 142 differentially expressed genes, respectively; of these, 80, 85, 92, 93, and 84 genes were upregulated and 58, 63, 61, 62, and 58 genes were downregulated, respectively (Figures 7A,B, Tables 3, 4). Overlap analysis of the differentially expressed genes showed that 103 genes were commonly shared by macrophages infected with all five strains (*P* < 0.05) (Figures 7A,B, Tables 3, 4). Comparison of macrophages infected with Δ*crp*-*cya* to those of its derivatives Δ*crp*-*cya*-Δ*phoPQ*, Δ*crp*-*cya*-Δ*sifA* and Δ*crp*-*cya*-Δ*sopF* identified 7, 3 and 1 differentially expressed genes (Supplementary Table 4).

**Table 3:** Genes upregulated in primary macrophages infected with *S*. Typhimurium UK1, Δ*crp*-*cya*, Δ*crp*-*cya*-Δ*phoPQ*, Δ*crp*-*cya*-Δ*sifA* and Δ*crp*-*cya*-Δ*sopF*.

**Functional category**	**Gene ID**	**Δ*crp*-*cya*^A^**	***P*** **value**	**UK1^A^**	***P*** **value**	**Δ*crp*-*cya*-*ΔphoPQ*^A^**	**P value**	**Δ*crp*-*cya*-*ΔsifA*^A^**	***P*** **value**	**Δ*crp*-*cya*-*ΔsopF*^A^**	***P*** **value**
Toll-like receptor signaling pathway	PIK3R3	2.4	9.2E-02	2.6	2.5E-01	2.5	6.2E-02	2.3	2.6E-01	2.8	6.2E-01
	CCL5	2.3	9.7E-02	2.8	4.2E-02	3.0	2.0E-01	2.6	3.2E-01	2.6	3.3E-02
	TRAF3	6.6	3.8E-02	10.8	3.7E-02	12.1	9.2E-03	9.7	2.2E-01	8.1	1.9E-01
	DNM2	3.7	6.4E-02	2.0	3.4E-01	3.1	2.7E-01	4.0	4.2E-03	2.6	6.7E-01
	IRF7	2.5	4.8E-01	4.2	8.0E-02	5.6	2.1E-01	3.9	3.9E-01	–	–
	CCL4	45.8	3.8E-01	72.5	2.6E-01	66.7	2.7E-02	68.7	1.9E-01	58.1	4.5E-02
	REL	2.9	5.0E-02	3.8	4.0E-02	3.8	1.0E-02	3.5	1.0E-01	3.5	1.0E-01
	TRAF2	–	–	2.1	7.4E-02	2.5	1.4E-01	2.2	7.9E-02	–	–
	RIPK2	2.5	5.2E-01	3.1	9.3E-02	3.3	3.2E-01	2.8	9.8E-02	2.9	3.4E-01
	BIRC3	5.4	4.0E-01	5.9	8.1E-02	7.8	6.4E-03	6.9	2.6E-01	4.2	6.6E-01
	FOS	6.9	5.1E-01	3.1	2.8E-01	3.0	6.6E-02	5.5	4.2E-01	6.9	7.3E-01
	IRAK2	–	–	2.3	3.4E-01	2.3	6.0E-04	–	–	–	–
	MAP2K3	–	–	–	–	–	–	2.4	1.0E-01	–	–
	PELI3	2.6	2.7E-01	–	–	2.4	8.1E-02	2.5	7.0E-02	2.4	6.9E-01
	IL8L1	52.0	3.0E-01	103.0	2.0E-01	159.0	9.0E-02	108.8	1.0E-01	57.1	5.0E-01
	TASL	10.0	1.0E-01	22.0	2.0E-01	30.0	6.0E-02	21.1	2.3E-01	7.5	6.0E-01
	DUSP4	2.5	5.0E-01	–	–	2.2	1.0E-01	2.8	6.0E-02	2.5	7.6E-01
	JUN	2.4	5.0E-01	–	–	2.0	7.0E-02	2.2	5.0E-02	2.3	7.0E-01
Cytokine-cytokine receptor interaction											
	CCL5	2.3	9.7E-02	2.8	4.2E-02	3.0	2.0E-01	2.6	3.2E-01	2.6	3.3E-02
	IL16	2.6	5.0E-02	2.3	2.5E-01	2.3	2.0E-02	2.3	3.0E-01	2.8	4.0E-01
	IL1B	71.0	1.0E-01	138.0	2.0E-01	134.0	2.0E-01	110.0	4.0E-01	88.0	7.0E-01
	IL1R2	3.9	2.0E-02	5.9	1.0E-01	6.7	2.0E-02	6.3	5.0E-02	4.4	2.0E-02
	CSF3	7.8	6.0E-02	21.0	3.0E-01	14.0	3.0E-01	13.0	5.0E-01	9.0	7.0E-01
	CD44	7.3	2.0E-02	8.6	1.0E-01	15.0	1.0E-01	9.5	4.0E-02	7.9	2.0E-01
	MCL1	2.1	4.0E-02	–	–	2.3	1.0E-01	2.3	3.0E-01	2.2	7.0E-01
	CCL4	45.8	3.8E-01	72.5	2.6E-01	66.7	2.7E-02	68.7	1.9E-01	58.1	4.5E-02
	IL10RA	2.9	2.0E-01	5.0	5.0E-02	5.5	1.0E-01	4.6	2.0E-01	2.9	6.0E-01
	REL	2.9	5.0E-02	3.8	4.0E-02	3.8	1.0E-02	3.5	1.0E-01	3.5	1.0E-01
	IRF7	2.5	4.0E-01	4.2	8.0E-02	5.6	2.0E-01	3.9	3.9E-01	–	–
	PTPN2	2.2	1.0E-01	2.5	6.0E-02	2.4	1.0E-01	2.4	1.0E-01	2.3	4.0E-01
	RIPK2	2.5	5.2E-01	3.1	9.3E-02	3.3	3.2E-01	2.8	9.8E-02	2.9	3.4E-01
	BIRC3	5.4	4.0E-01	5.9	8.1E-02	7.8	6.4E-03	6.9	2.6E-01	4.2	6.6E-01
	TRAF2	–	–	2.1	7.4E-02	2.5	1.4E-01	2.2	7.9E-02	–	–
	TRAF3	6.6	3.8E-02	10.8	3.7E-02	12.1	9.2E-03	9.7	2.2E-01	8.1	1.9E-01
	PELI3	2.6	2.7E-01	–	–	2.4	8.1E-02	2.5	7.0E-02	2.4	6.9E-01
	IFNLR1	2.1	4.6E-01	–	–	2.5	7.0E-02	2.7	3.0E-01	2.2	7.0E-01
	IRAK2	–	–	2.3	3.4E-01	2.3	6.0E-04	–	–	–	–
	MAP2K3	–	–	–	–	–	–	2.4	1.0E-01	–	–
	EGR1	7.4	4.0E-01	4.5	3.0E-01	6.6	2.0E-02	6.5	1.0E-01	7.2	7.0E-01
	PIK3R3	2.4	9.2E-02	2.6	2.5E-01	2.5	6.2E-02	2.3	2.6E-01	2.8	6.2E-01
	FOS	6.9	5.1E-01	3.1	2.8E-01	3.0	6.6E-02	5.5	4.2E-01	6.9	7.3E-01
	TNFRSF6B	19.0	4.0E-01	33.0	1.0E-01	19.4	5.0E-02	29.0	4.0E-01	25.0	7.0E-01
	CCL1	3.1	4.0E-01	8.1	3.0E-01	12.0	4.0E-02	7.0	2.0E-01	3.1	7.0E-01
	IL8L1	52.0	3.0E-01	103.0	2.0E-01	159.0	9.0E-02	108.8	1.0E-01	57.1	5.0E-01
	TNFRSF6B	19.0	4.0E-01	33.0	1.0E-01	19.0	5.0E-02	29.0	4.0E-01	25.0	7.0E-01
	GAB2	8.1	3.0E-01	–	–	–	–	2.3	7.0E-02	3.8	7.0E-01
	BCL6	2.8	4.0E-01	2.5	2.0E-01	3.0	1.0E-01	3.0	4.0E-02	2.6	7.0E-01
	TNFRSF9	–	–	18.2	2.0E-01	3.7	3.0E-01	4.5	5.0E-02	5.2	7.0E-01
	DUSP4	2.5	5.0E-01	–	–	2.2	1.0E-01	2.8	6.0E-02	2.5	7.6E-01
	IL18	3.5	3.0E-01	6.9	2.0E-01	7.5	2.0E-01	5.1	2.0E-02	5.3	7.0E-01
	JUN	2.4	5.0E-01	–	–	2.0	7.0E-02	2.2	5.0E-02	2.3	7.0E-01
	CSF2	20.6	2.0E-01	31.0	4.0E-01	19.0	2.5E-01	18.8	4.0E-02	16.8	7.0E-01
	HIVEP2	–	–	–	–	2.3	5.0E-02	2.1	2.0E-02	2.1	5.0E-01
	TRIB2	3.0	3.0E-01	2.5	1.0E-02	2.5	2.0E-02	2.6	2.0E-02	3.1	8.0E-03
	PELI3	2.6	2.7E-01	2.1	1.0E-01	2.4	8.1E-02	2.5	7.0E-02	2.4	6.9E-01
MAP kinase pathway											
	TRAF3	6.6	3.8E-02	10.8	3.7E-02	12.1	9.2E-03	9.7	2.2E-01	8.1	1.9E-01
	HBEGF	9.8	1.0E-02	19.0	2.0E-01	18.0	2.0E-01	16.3	4.0E-01	13.5	7.0E-01
	DUSP10	2.1	3.0E-02	–	–	–	–	–	–	2.1	6.0E-01
	RASGEF1A	31.9	1.0E-03	35.5	1.0E-01	52.1	1.0E-01	42.2	2.0E-01	13.0	4.0E-01
	TRIB2	3.0	3.0E-01	2.5	1.0E-02	2.5	2.0E-02	2.6	2.0E-02	3.1	8.0E-03
	APBB1IP	2.1	4.0E-01	–	–	2.3	9.0E-02	2.3	1.0E-01	2.4	7.0E-01
	CDC14B	–	–	12.8	1.0E-01	2.8	1.0E-02	2.7	6.0E-01	–	–
	DUSP8	5.2	4.0E-01	4.7	1.0E-01	6.0	6.0E-02	6.1	1.0E-01	5.1	6.0E-01
	DUSP5	7.4	4.0E-01	6.7	2.0E-01	10.7	2.0E-01	9.0	1.0E-02	7.2	6.0E-01
	RIPK2	2.5	5.2E-01	3.1	9.3E-02	3.3	3.2E-01	2.8	9.8E-02	2.9	3.4E-01
	DUSP4	2.5	5.0E-01	–	–	2.2	1.0E-01	2.8	6.0E-02	2.5	7.6E-01
	JUN	2.4	5.0E-01	–	–	2.0	7.0E-02	2.2	5.0E-02	2.3	7.0E-01
	CSF2	20.6	2.0E-01	31.0	4.0E-01	19.0	2.5E-01	18.8	4.0E-02	16.8	7.0E-01
Organelle biogenesis											
	HELZ2	16.2	5.0E-03	10.8	1.0E-01	8.0	3.0E-01	12.8	4.0E-01	17.1	4.0E-01
	GABPB1	3.0	6.0E-02	2.0	1.0E-01	3.8	8.0E-02	4.2	4.0E-02	3.4	7.0E-01
	DNM2	3.7	6.4E-02	2.0	3.4E-01	3.1	2.7E-01	4.0	4.2E-03	2.6	6.7E-01
Regulation of gene expression											
	GABPB1	3.0	6.0E-02	2.0	1.0E-01	3.8	8.0E-02	4.2	4.0E-02	3.4	7.0E-01
	CREM	2.1	9.0E-03	–	–	–	–	–	–	–	–
	RDH10	2.7	3.0E-02	3.2	3.0E-01	3.0	2.0E-01	2.8	3.0E-01	3.5	5.0E-01
	CITED2	4.0	1.0E-02	3.0	5.0E-01	–	–	3.4	4.0E-01	3.7	7.0E-01
	F3	79.0	4.0E-01	148.0	1.0E-02	143.0	2.0E-01	102.0	2.0E-01	109.0	7.0E-01
	ARC	11.0	1.0E-01	22.0	9.0E-02	18.0	2.0E-01	16.2	2.0E-01	15.0	8.0E-02
	BTG2	5.2	4.0E-01	5.4	5.0E-02	6.3	8.0E-02	6.4	1.0E-01	5.9	5.0E-01
	ZBTB21	2.1	1.0E-01	2.3	4.0E-02	2.8	2.0E-01	2.5	3.0E-01	2.5	5.0E-01
	KLF5	2.3	3.0E-01	2.2	6.0E-02	2.4	5.0E-02	2.4	2.0E-01	3.0	3.0E-02
	CREB5	25.3	2.0E-01	4.6	5.0E-02	9.6	4.0E-01	4.6	4.0E-01	7.0	7.0E-02
	FOS	6.9	5.1E-01	3.1	2.8E-01	3.0	6.6E-02	5.5	4.2E-01	6.9	7.3E-01
	EGR1	7.4	4.0E-01	4.5	3.0E-01	6.6	2.0E-02	6.5	1.0E-01	7.2	7.0E-01
	SGK1	3.2	3.0E-01	3.2	1.0E-01	3.9	4.0E-02	3.6	1.0E-01	3.4	6.0E-01
	SNAI1	2.3	5.0E-01	3.1	4.0E-01	4.5	4.0E-02	3.0	1.0E-01	–	–
	CSRNP1	3.0	4.0E-01	3.2	1.0E-01	3.7	4.0E-02	3.5	8.0E-02	3.1	1.0E-01
	KLF11	2.5	4.0E-01	–	–	2.5	9.0E-02	2.8	1.0E-01	2.4	7.0E-01
	H3F3B	2.2	5.0E-01	–	–	2.2	7.0E-02	2.4	2.0E-01	2.1	7.0E-01
	SP4	13.3	4.0E-01	5.5	3.0E-01	13.6	3.0E-02	14.8	2.0E-01	30.0	7.0E-01
	PHF19	–	–	–	–	2.1	4.0E-02	2.5	3.0E-01	–	–
	FSBP	3.7	5.0E-01	5.8	2.0E-01	8.3	8.0E-02	4.9	1.0E-01	3.3	4.0E-01
	CITED4	3.1	4.0E-01	5.2	2.0E-01	5.9	5.0E-02	5.0	3.0E-01	3.0	6.0E-01
	PPM1D	–	–	–	–	2.1	5.0E-02	2.2	1.0E-01	–	–
	KLF6	3.4	3.0E-01	2.7	1.0E-01	3.3	6.0E-02	3.1	2.0E-02	3.2	6.0E-01
	HIVEP2	–	–	–	–	2.3	5.0E-02	2.1	2.0E-02	2.1	5.0E-01
	NR4A3	7.3	4.0E-01	5.8	1.0E-01	8.2	1.0E-01	8.5	1.0E-02	9.6	1.0E-02
	MAMLD1	–	–	–	–	2.3	1.0E-01	2.2	8.0E-02	–	–
	JUN	2.4	5.0E-01	–	–	2.0	7.0E-02	2.2	5.0E-02	2.3	7.0E-01
	RRAD	5.4	3.0E-01	4.6	1.0E-01	4.7	1.0E-01	4.4	1.0E-01	5.4	2.0E-02
	ID2	2.6	4.0E-01	2.1	1.0E-01	2.2	1.0E-01	2.4	3.0E-01	2.5	2.0E-02
GPCR signaling pathway											
	CCL5	2.3	9.7E-02	2.8	4.2E-02	3.0	2.0E-01	2.6	3.2E-01	2.6	3.3E-02
	HBEGF	9.8	1.0E-02	19.0	2.0E-01	18.0	2.0E-01	16.3	4.0E-01	13.5	7.0E-01
	PIK3R3	2.4	9.2E-02	2.6	2.5E-01	2.5	6.2E-02	2.3	2.6E-01	2.8	6.2E-01
	GPR183	–	–	4.4	2.0E-03	4.5	1.0E-01	3.0	3.0E-01	–	–
	BDKRB1	35.0	3.0E-01	50.0	3.0E-02	40.7	2.0E-01	41.2	3.0E-01	35.7	7.0E-01
	CCLL4	2.3	3.0E-01	2.1	7.0E-02	–	–	–	–	2.4	7.0E-01
	RGS1	3.6	4.0E-01	2.9	7.0E-02	3.6	8.0E-02	3.9	1.0E-01	3.6	7.0E-01
	IL8L1	52.0	3.0E-01	103.0	2.0E-01	159.0	9.0E-02	108.8	1.0E-01	57.1	5.0E-01
	GPR68	3.3	3.0E-01	2.8	3.0E-01	3.4	7.0E-02	3.7	2.0E-01	3.2	7.0E-01
	PIK3R5	–	–	2.9	1.0E-01	2.9	8.0E-02	2.3	3.0E-01	–	–
	RGS3	4.9	2.0E-01	96.7	1.0E-01	96.7	9.0E-03	96.7	3.0E-01	5.5	1.0E-01
	CCL1	3.1	4.0E-01	8.1	3.0E-01	12.0	4.0E-02	7.0	2.0E-01	3.1	7.0E-01
	PRKCA	3.0	4.0E-01	3.0	3.0E-01	3.2	8.0E-02	3.3	3.0E-01	2.4	7.0E-01
	PDE4B	–	–	2.3	3.0E-01	2.7	9.0E-02	2.6	3.0E-01	2.4	2.0E-01
	CCL4	45.8	3.8E-01	72.5	2.6E-01	66.7	2.7E-02	68.7	1.9E-01	58.1	4.5E-02
	RGS9	2.6	3.0E-01	2.5	2.0E-01	3.7	1.0E-01	3.5	4.0E-02	2.8	7.0E-01
	CGNRH-R	3.2	5.0E-01	5.1	3.0E-01	5.6	4.0E-01	4.1	3.0E-01	3.7	4.0E-02
NOD-like receptor signaling pathway											
	CCL5	2.3	9.7E-02	2.8	4.2E-02	3.0	2.0E-01	2.6	3.2E-01	2.6	3.3E-02
	TRAF3	6.6	3.8E-02	10.8	3.7E-02	12.1	9.2E-03	9.7	2.2E-01	8.1	1.9E-01
	RIPK2	2.5	5.2E-01	3.1	9.3E-02	3.3	3.2E-01	2.8	9.8E-02	2.9	3.4E-01
	BIRC3	5.4	4.0E-01	5.9	8.1E-02	7.8	6.4E-03	6.9	2.6E-01	4.2	6.6E-01
	TRAF2	–	–	2.1	7.4E-02	2.5	1.4E-01	2.2	7.9E-02	–	–
	IRF7	2.5	4.8E-01	4.2	8.0E-02	5.6	2.1E-01	3.9	3.9E-01	–	–
	IL8L1	52.0	3.0E-01	103.0	2.0E-01	159.0	9.0E-02	108.8	1.0E-01	57.1	5.0E-01
	JUN	2.4	5.0E-01	–	–	2.0	7.0E-02	2.2	5.0E-02	2.3	7.0E-01
	IL18	3.5	3.0E-01	6.9	2.0E-01	7.5	2.0E-01	5.1	2.0E-02	5.3	7.0E-01
Apoptosis											
	CITED2	4.0	1.0E-02	3.0	5.0E-01	–	–	3.4	4.0E-01	3.7	7.0E-01
	BIRC3	5.4	4.0E-01	5.9	8.1E-02	7.8	6.4E-03	6.9	2.6E-01	4.2	6.6E-01
	TRAF2	–	–	2.1	7.4E-02	2.5	1.4E-01	2.2	7.9E-02	–	–
	PLEKHN1	2.5	4.0E-01	2.4	7.0E-02	2.6	1.0E-02	3.3	1.0E-01	2.4	7.0E-01
	CFLAR	–	–	2.1	1.0E-01	3.0	2.0E-02	2.5	3.0E-01	–	–
	BIRC3	5.4	4.0E-01	5.9	8.1E-02	7.8	6.4E-03	6.9	2.6E-01	4.2	6.6E-01
	PMAIP1	31.0	4.0E-01	18.0	2.0E-01	14.0	8.0E-02	32.0	4.0E-01	36.0	6.0E-01
	PIK3R3	2.4	9.2E-02	2.6	2.5E-01	2.5	6.2E-02	2.3	2.6E-01	2.8	6.2E-01
	FOS	6.9	5.1E-01	3.1	2.8E-01	3.0	6.6E-02	5.5	4.2E-01	6.9	7.3E-01
	ZC3H12A	7.5	2.0E-01	8.9	5.0E-03	8.9	1.0E-02	8.5	1.0E-01	7.9	2.0E-01
	PPIF	4.7	4.0E-01	4.8	1.0E-01	4.1	1.0E-02	3.9	1.0E-02	4.8	4.0E-01
	ZFAND5	2.2	3.0E-01	–	–	–	–	2.1	4.0E-02	2.0	7.0E-01
	BCL6	2.8	4.0E-01	2.5	2.0E-01	3.0	1.0E-01	3.0	4.0E-02	2.6	7.0E-01
	JUN	2.4	5.0E-01	–	–	2.0	7.0E-02	2.2	5.0E-02	2.3	7.0E-01
	PHLDA2	3.5	1.0E-01	3.4	5.0E-02	3.6	2.0E-01	3.4	3.0E-01	3.3	7.0E-02
Host-microbe interaction											
	GUCY2C	2.1	6.0E-02	2.4	4.0E-01	2.7	2.0E-01	–	–	–	–
	HBEGF	9.8	1.0E-02	19.0	2.0E-01	18.0	2.0E-01	16.3	4.0E-01	13.5	7.0E-01
	CCL4	45.8	3.8E-01	72.5	2.6E-01	66.7	2.7E-02	68.7	1.9E-01	58.1	4.5E-02
	FOS	6.9	5.1E-01	3.1	2.8E-01	3.0	6.6E-02	5.5	4.2E-01	6.9	7.3E-01
	IL8L1	52.0	3.0E-01	103.0	2.0E-01	159.0	9.0E-02	108.8	1.0E-01	57.1	5.0E-01
	MYO9B	–	–	2.1	1.0E-01	2.5	8.0E-02	2.2	8.0E-02	–	–
	JUN	2.4	5.0E-01	–	–	2.0	7.0E-02	2.2	5.0E-02	2.3	7.0E-01
Cytosolic DNA-sensing pathway											
	CCL5	2.3	9.7E-02	2.8	4.2E-02	3.0	2.0E-01	2.6	3.2E-01	2.6	3.3E-02
	IRF7	2.5	4.8E-01	4.2	8.0E-02	5.6	2.1E-01	3.9	3.9E-01	–	–
	CCL4	45.8	3.8E-01	72.5	2.6E-01	66.7	2.7E-02	68.7	1.9E-01	58.1	4.5E-02
	IL18	3.5	3.0E-01	6.9	2.0E-01	7.5	2.0E-01	5.1	2.0E-02	5.3	7.0E-01
NF-kB pathway											
	REL	2.9	5.0E-02	3.8	4.0E-02	3.8	1.0E-02	3.5	1.0E-01	3.5	1.0E-01
	BIRC3	5.4	4.0E-01	5.9	8.1E-02	7.8	6.4E-03	6.9	2.6E-01	4.2	6.6E-01
	TRAF2	–	–	2.1	7.4E-02	2.5	1.4E-01	2.2	7.9E-02	–	–
	TRAF3	6.6	3.8E-02	10.8	3.7E-02	12.1	9.2E-03	9.7	2.2E-01	8.1	1.9E-01
	PIM3	3.7	4.0E-01	2.7	7.0E-02	3.1	1.0E-01	3.4	1.0E-01	3.5	5.0E-01
	ZC3H12A	7.5	2.0E-01	8.9	5.0E-03	8.9	1.0E-02	8.5	1.0E-01	7.9	2.0E-01
	TNFRSF6B	19.0	4.0E-01	33.0	1.0E-01	19.4	5.0E-02	29.0	4.0E-01	25.0	7.0E-01
	ZFAND5	2.2	3.0E-01	–	–	–	–	2.1	4.0E-02	2.0	7.0E-01
	RIPK2	2.5	5.2E-01	3.1	9.3E-02	3.3	3.2E-01	2.8	9.8E-02	2.9	3.4E-01
	TNFRSF9	–	–	18.2	2.0E-01	3.7	3.0E-01	4.5	5.0E-02	5.2	7.0E-01
	PELI3	2.6	2.7E-01	–	–	2.4	8.1E-02	2.5	7.0E-02	2.4	6.9E-01
Cytoskeleton remodeling											
	TLN2	2.4	2.0E-01	3.4	4.0E-02	5.6	1.0E-01	3.5	4.0E-02	2.2	1.0E-01
	MOB3C	2.7	5.0E-01	3.6	5.0E-02	5.4	7.0E-02	4.5	2.0E-02	2.8	7.0E-01
	FAM83H	2.8	4.0E-01	–	–	2.9	1.0E-01	3.1	7.0E-02	3.1	7.0E-01
	RAI14	2.1	5.0E-01	–	–	–	–	–	–	2.3	5.0E-02
Metabolism											
	DCTN2	2.6	5.0E-01	3.6	6.0E-01	7.3	6.0E-02	6.2	4.0E-01	4.4	7.0E-01
	HDC	3.6	5.0E-01	4.3	1.0E-01	3.1	8.0E-02	2.4	5.0E-01	4.5	7.0E-01
	ODC1	2.3	4.0E-01	2.1	2.0E-01	2.7	9.0E-02	2.8	3.0E-01	2.3	7.0E-01
	PFKFB2	–	–	–	–	3.1	5.0E-02	–	–	–	–
	CHST9	–	–	–	–	3.2	6.0E-02	3.4	4.0E-01	–	–
	KLF5	2.3	3.0E-01	2.2	6.0E-02	2.4	5.0E-02	2.4	2.0E-01	3.0	3.0E-02
	PDP1	2.2	3.0E-01	–	–	–	–	–	–	2.1	8.0E-02

A *Fold change in gene expression in macrophages infected with S. Typhimurium UK1, Δcrp-cya, Δcrp-cya-ΔphoPQ, Δcrp-cya-ΔsifA and Δcrp-cya-ΔsopF compared to uninfected macrophages*.

**Table 4:** Genes downregulated in primary macrophages infected with *S*. Typhimurium UK1, Δ*crp-cya*, Δ*crp-cya-*Δ*phoPQ*, Δ*crp-cya-*Δ*sifA* and Δ*crp-cya-*Δ*sopF*.

**Functional category**	**Gene ID**	**Δ*crp*-*cya*^A^**	***P*** **value**	**UK1^A^**	***P*** **value**	**Δ*crp*−*cya*−*ΔphoPQ*^A^**	***P*** **value**	**Δ*crp*−*cya*−*ΔsifA*^A^**	***P*** **value**	**Δ*crp*−*cya*−*ΔsopF*^A^**	***P*** **value**
Cell cycle											
	HAUS3	2.3	4.0E-02	2.9	1.0E-01	2.1	1.0E-01	2.1	3.0E-01	–	–
	NEK2	2.6	7.0E-02	2.6	6.0E-02	2.6	9.0E-02	2.3	1.0E-01	2.4	1.0E-02
	DNA2	3.8	1.0E-03	2.8	3.0E-01	2.3	1.0E-01	2.9	2.0E-03	3.1	1.0E-01
	CCNE2	2.6	8.0E-02	3	1.0E-01	–	–	–	–	2.9	5.0E-01
	NCAPG2	2.7	7.0E-02	2.5	1.0E-01	–	–	2.7	1.0E-01	2.7	5.0E-01
	RRM2	2.7	4.0E-02	–	–	201	1.0E-01	2.2	5.0E-02	2.3	1.0E-01
	SMC2	3.3	9.0E-02	2.6	2.0E-01	2.7	1.0E-01	2.9	2.0E-01	3	2.0E-01
	SMC4	2.9	1.0E-01	2.2	1.0E-01	–	–	2.1	3.0E-01	2.2	6.0E-01
	CCNB3	2.5	3.0E-02	–	–	–	–	–	–	2.6	5.0E-02
	CHD2	2.1	1.0E-01	2	1.0E-02	2.2	2.0E-02	2.1	5.0E-02	2.1	4.0E-01
	MST1	2.6	3.0E-01	2.5	1.0E-02	3	2.0E-01	3.3	2.0E-01	–	–
	MRNIP	–	–	–	–	2.1	8.0E-02	2.4	1.0E-01	–	–
Post-translational protein modification											
	ANKRD9	2.1	6.0E-02	2.5	4.0E-02	2.5	2.0E-01	2.1	4.0E-01	–	–
	MAN1C1	2.2	5.0E-02	–	–	2.2	4.0E-02	2.1	7.0E-02	–	–
	KLHL25	3.8	4.0E-02	3.5	1.0E-01	4.2	4.0E-02	3.9	1.0E-01	3.4	7.0E-02
	SOCS2	3.5	4.0E-01	4	3.0E-01	3.9	2.0E-01	2.6	3.0E-01	2.2	4.0E-01
	TRAIP	2.5	4.0E-01	5.1	2.0E-02	–	–	2.4	1.0E-01	2.1	6.0E-01
	RNF208	4.8	4.0E-01	6.1	2.0E-01	12.7	1.0E-01	8	4.0E-03	5	4.0E-01
	PRKDC	2	1.0E-01	2.1	9.0E-02	–	–	2.1	6.0E-02	–	–
Cellular senescence											
	CCNE2	2.6	8.0E-02	3	1.0E-01	–	–	–	–	2.9	5.0E-01
	RASSF5	8.8	1.0E-02	17.8	2.0E-01	20.4	2.0E-01	15.6	5.0E-01	5.3	2.0E-01
	CCNB3	2.5	3.0E-02	–	–	–	–	–	–	2.6	5.0E-02
	PIK3CD	2.3	9.0E-02	2.2	1.0E-01	2.1	1.0E-01	–	–	–	–
	BHLHA15	2.7	5.0E-01	2.6	2.0E-01	5.9	9.0E-02	2.2	2.0E-01	2.7	7.0E-01
Transport of small molecules and ions											
	TRPV2	2.6	4.0E-02	–	–	5.4	1.0E-01	–	–	–	–
	TTYH2	2.4	4.0E-02	2.5	4.0E-02	3.1	4.0E-02	2.7	1.0E-01	2.3	6.0E-01
	TTYH3	21	8.0E-02	9.8	1.0E-01	8.7	9.0E-02	4.3	2.0E-01	6.3	3.0E-01
	SLC17A5	16.1	6.0E-02	5.6	3.0E-01	2.9	4.0E-01	6.7	1.0E-01	7.4	5.0E-01
	ABCG2	3.3	5.0E-02	3	1.0E-01	3.4	2.0E-02	3.3	4.0E-02	3.2	3.0E-01
	ABCC5	2.2	1.0E-02	2.4	1.0E-01	2.5	1.0E-01	2.3	3.0E-01	2.3	8.0E-02
	SLC29A3	–	–	2.3	5.0E-02	2.1	1.0E-01	–	–	2.1	4.0E-01
	LIPC	2.5	2.0E-01	2.2	3.0E-02	–	–	–	–	–	–
	NIPAL1	4.2	3.0E-01	3.9	5.0E-02	3.1	1.0E-01	2.5	2.0E-01	3.2	5.0E-01
	SLC38A7	–	–	2.2	4.0E-02	–	–	–	–	–	–
	SLC6A6	3.1	2.0E-01	2.6	3.0E-01	3.5	3.0E-02	3.8	2.0E-01	3.9	7.0E-01
	PKD2L2	4.1	1.0E-01	3.3	1.0E-01	3.3	6.0E-02	3.9	7.0E-02	4.3	3.0E-02
	TMC5	2.4	4.0E-01	2.7	2.0E-01	5.1	6.0E-02	3.4	2.0E-01	2.5	6.0E-01
	SYNGR3	3.1	3.0E-01	6	3.0E-01	2.6	1.0E-02	3.5	3.0E-01	2.9	6.0E-01
	SLC7A5	2.3	3.0E-01	2.1	4.0E-03	2.7	6.0E-02	2.4	3.0E-01	2.2	6.0E-01
	SCARB1	4.6	1.0E-01	11	1.0E-01	9.6	1.0E-01	5.9	5.0E-02	3.4	7.0E-01
ABC transporters											
	ABCC5	2.2	1.0E-02	2.4	1.0E-01	2.5	1.0E-01	2.3	3.0E-01	2.3	8.0E-02
	ABCG2	3.3	5.0E-02	3	1.0E-01	3.4	2.0E-02	3.3	4.0E-02	3.2	3.0E-01
	ABCG1	–	–	–	–	2.1	9.0E-02	2.2	1.0E-02	–	–
Carbohydrate metabolism											
	FUT7	3.5	3.0E-02	2.7	2.0E-01	3.2	1.0E-01	4.3	2.0E-01	2.1	7.0E-01
	GALE	7.7	9.0E-02	–	–	3.4	2.0E-01	2.6	5.0E-01	2.7	6.0E-01
	GNE	–	–	2.2	8.0E-02	–	–	–	–	–	–
	HEXD	2.2	2.0E-01	2.6	7.0E-02	2.2	1.0E-01	2.2	2.0E-01	2	1.0E-01
	PDK2	–	–	–	–	2.3	3.0E-02	–	–	–	–
	FBP1	2.3	4.0E-01	–	–	2.5	2.0E-01	2.7	6.0E-03	2.2	6.0E-01
	XYLT1	4	5.0E-01	–	–	–	–	–	–	4	1.0E-02
Signal transduction											
	ARAP3	2.7	7.0E-02	2.2	7.0E-02	–	–	2.1	2.0E-01	2.1	1.0E-01
	ARHGAP25	2.7	7.0E-02	2.5	1.0E-01	2.5	7.0E-02	2.4	6.0E-02	2	6.0E-02
	RACGAP1	2.2	4.0E-03	2.4	3.0E-01	–	–	2.1	1.0E-01	3.2	5.0E-01
	LFNG	2.1	8.0E-02	2.3	7.0E-02	2.4	3.0E-02	2.1	5.0E-02	2.1	2.0E-01
	MOSMO	5.5	6.0E-02	–	–	2.8	2.0E-01	–	–	3	3.0E-01
	CXCR4	3.8	9.0E-02	4.3	1.0E-01	3.7	5.0E-02	3.8	6.0E-02	4.4	5.0E-02
	PIK3CD	2.3	9.0E-02	2.2	1.0E-01	2.1	1.0E-01	–	–	–	–
	MST1	2.6	3.0E-01	2.5	1.0E-02	3	2.0E-01	3.3	2.0E-01	–	–
	NLRX1	2.2	1.0E-01	2.6	8.0E-02	–	–	–	–	2.6	3.0E-01
	ENSGALT00000095967	–	–	2.3	9.0E-02	2.1	3.0E-01	2.1	2.0E-01	2.1	5.0E-01
	TTC3	2.9	4.0E-01	3.1	2.0E-01	2.1	4.0E-01	2.7	2.0E-01	4.1	9.0E-02
	PRAG1	2.5	3.0E-01	2.3	2.0E-01	2.1	2.0E-01	–	–	2.4	6.0E-02
	ARHGEF39	3	2.0E-01	–	–	–	–	2.9	3.0E-01	5.2	8.0E-02
	ARHGAP25	2.7	7.0E-02	2.5	1.0E-01	2.5	7.0E-02	2.4	6.0E-02	2.6	6.0E-02
	FLOT2	2.1	4.0E-01	3.9	8.0E-02	5.5	5.0E-02	2.9	2.0E-01	3.4	5.0E-02
p53 signaling pathway											
	CCNE2	2.6	8.0E-02	3	1.0E-01	–	–	–	–	2.9	5.0E-01
	RRM2	2.7	4.0E-02	–	–	201	1.0E-01	2.2	5.0E-02	2.3	1.0E-01
	SESN2	2.2	5.0E-01	–	–	–	–	2.2	3.0E-01	–	–
Lipid metabolism											
	ACACA	2.1	9.0E-02	–	–	2.4	2.0E-01	2.5	9.0E-02	–	–
	FUT7	3.5	3.0E-02	2.7	2.0E-01	3.2	1.0E-01	4.3	2.0E-01	2.11	7.0E-01
	DPEP2	2.7	9.0E-02	2.1	1.0E-01	2.1	1.0E-01	2.5	2.0E-01	2.5	5.0E-01
	TNFAIP8	4.2	2.0E-02	3.4	6.0E-02	2.6	2.0E-01	2.3	1.0E-01	2.6	3.0E-01
	HMGCL	2.5	2.0E-01	2.7	6.0E-02	3.01	6.0E-02	3.3	3.0E-01	6.1	3.0E-01
	PLA2G15	2.5	3.0E-01	2.2	6.0E-02	3.2	5.0E-02	3.2	9.0E-02	2.4	6.0E-01
	GSTA3	–	–	–	–	2.1	3.0E-02	2.1	4.0E-02	–	–
	ALOX5AP	2.3	2.0E-01	–	–	2.3	4.0E-02	2.23	1.0E-01	2	7.0E-01
	GLB1L	–	–	–	–	2.3	4.0E-02	2.1	4.0E-01	–	–
	THEM4	–	–	–	–	3.1	9.0E-02	–	–	–	–
	PLEKHA2	2.4	2.0E-01	3	8.0E-02	2.7	7.0E-02	2.3	1.0E-01	2.7	1.0E-01
	PLA2G15	2.5	3.0E-01	2.2	6.0E-02	3.25	5.0E-02	3.2	9.0E-02	2.4	6.0E-01
	TBXAS1	2.4	2.0E-01	2.2	3.0E-01	2.8	1.0E-01	2.5	3.0E-02	2.1	7.0E-01
	ACOX2	–	–	2.6	3.0E-01	–	–	2.5	5.0E-02	–	–
	UGT8	2.3	2.0E-01	2.1	1.0E-01	2.1	1.0E-01	2.1	5.0E-02	2.5	5.0E-02
	EFR3B	–	–	2.2	2.0E-02	2.2	2.0E-01	–	–	2.1	9.0E-02
	PI4KB	–	–	2.3	5.0E-01	–	–	–	–	2.5	5.0E-02
Autophagy											
	PIK3CD	2.3	9.0E-02	2.2	1.0E-01	2.1	1.0E-01	–	–	–	–
	ULK2	2.2	7.0E-02	2.2	1.0E-01	2.1	4.0E-02	2.2	1.0E-01	2.2	6.0E-03
	RASSF5	8.8	1.0E-02	17.8	2.0E-01	20.4	2.0E-01	15.6	5.0E-01	5.3	2.0E-01
	FLOT2	2.1	4.0E-01	3.9	8.0E-02	5.5	5.0E-02	2.9	2.0E-01	3.4	5.0E-02
Amino acid metabolism											
	SLC7A5	2.3	3.0E-01	2.1	4.0E-03	2.7	6.0E-02	2.4	3.0E-01	2.2	6.0E-01
	KMO	3.2	3.0E-01	2.2	7.0E-02	3.6	1.4E-01	4.1	2.0E-02	2.4	7.0E-01
	GLB1L	–	–	–	–	2.3	4.0E-02	2.1	4.0E-01	–	–
	ENSGALT00000089357	–	–	–	–	2.3	9.0E-02	2.1	9.0E-02	2.1	5.0E-01
	CAT	–	–	–	–	2.2	1.0E-01	2.2	5.0E-02	–	–

A *Fold change in gene expression in macrophages infected with S. Typhimurium UK1, Δcrp-cya, Δcrp-cya-ΔphoPQ, Δcrp-cya-ΔsifA and Δcrp-cya-ΔsopF compared to uninfected macrophages*.

Pathway enrichment analyses showed that the following pathways were significantly enriched among the genes upregulated in macrophages infected with UK1 (*P* < 0.05): cytokine-cytokine receptor interaction, NOD-like receptor signaling pathway, Toll-like receptor signaling pathway, influenza A, AGE-RAGE signaling pathway in diabetic complications, herpes simplex infection, *Salmonella* infection, cytosolic DNA-sensing pathway, apoptosis, RIG-I-like receptor signaling pathway, GnRH signaling pathway, necroptosis, MAPK signaling pathway and ErbB signaling pathway (*P* <0.05) (Supplementary Table 2). Similar pathways were significantly enriched among the genes upregulated in macrophages infected with Δ*crp*-*cya*, Δ*crp*-*cya*-Δ*phoPQ*, Δ*crp*-*cya*-Δ*sifA* and Δ*crp*-*cya*-Δ*sopF* strains (*P* < 0.05) (Supplementary Table 2).

Among the downregulated genes, the following pathways were significantly enriched in macrophages infected with the five strains (*P* < 0.05): insulin signaling pathway, various metabolic pathways, cellular senescence and ABC transporters (Supplementary Table 3).

## Discussion

Serovars belonging to *Salmonella enterica* subspecies *enterica*, particularly *S*. Enteritidis and *S*. Typhimurium, are one of the most common causes of foodborne illnesses worldwide, with the majority of the illnesses attributed to consumption of contaminated poultry meat and eggs. Vaccination has been proven to be an effective strategy to reduce *Salmonella* burden in poultry. The *S*. Typhimurium *crp*-*cya* mutant is one of the widely used vaccines in the United States; however, the infection phenotypes and the early macrophage response to this vaccine strain are relatively poorly understood. Here, we characterized the infection phenotypes and the transcriptome response of macrophages to the vaccine strain and its derivatives lacking key genes required for intra-macrophage survival. Compared to the parent strain UK1, Δ*crp*-*cya* and its derivatives Δ*crp*-*cya*-Δ*phoPQ*, Δ*crp*-*cya*-Δ*ompR-envZ*, Δ*crp*-*cya*-Δ*feoABC* and Δ*crp*-*cya*-Δ*sifA* had greater doubling times in SCV-simulating media and were highly attenuated for invasion and intracellular survival within macrophages; the derivatives were more attenuated than Δ*crp*-*cya* mutant. The Δ*crp*-*cya* derivatives lacking *ssrAB, SPI13, SPI2, mgtRBC, sitABCD, sopF, sseJ* and *sspH2* had greater doubling times but surprisingly showed increased invasion and intracellular survival compared to the Δ*crp*-*cya* mutant. Transcriptome analyses of macrophages infected with parent strain, Δ*crp*-*cya* mutant and its derivatives lacking *phoPQ, sifA*, and *sopF* demonstrated that similar changes in gene expression were observed in macrophages infected with these strains. The differentially upregulated genes primarily belonged to innate immunity, immunoregulation, cellular homeostasis, and response to pathogens.

Cyclic AMP receptor protein (CRP) and adenylate cyclase (Cya) are two global regulators required for bacterial response to carbon starvation and these genes have been deleted in the Δ*crp*-*cya* mutant (Hassan and Curtiss, 1990). When grown in minimal media containing glucose as the sole carbon source, compared to UK1, the Δ*crp*-*cya* mutant and the majority of its derivatives exhibited increased doubling times. Interestingly, the vaccine strain derivatives Δ*crp*-*cya*-Δ*ompR*-*envZ*, Δ*crp*-*cya*-Δ*sopF*, and Δ*crp*-*cya*-Δ*sspH2* showed decreased doubling times than the Δ*crp*-*cya* strain but similar doubling times as the parent strain. Surprisingly, Δ*crp*-*cya*-Δ*phoPQ* strain had decreased doubling time than both the Δ*crp*-*cya* mutant and its parent strain. The reasons for these unusual phenotypes are unknown.

Consistent with the fact that the carbon starvation response genes *crp* and *cya* are deleted in the vaccine strain, the Δ*crp*-*cya* mutant and its derivatives exhibited significant defects in carbon source utilization. As expected, the parent strain demonstrated the ability to utilize a wide range of carbon sources. However, the Δ*crp*-*cya* mutant and its derivative were unable to metabolize the majority of carbon sources related to TCA cycle. All the mutants strains also exhibited significant growth defects in BHI broth without the added glucose (data not shown). A functional TCA cycle is required for *S*. Typhimurium virulence, and growth and survival within phagocytic cells (Yimga et al., 2006; Mercado-Lubo et al., 2009). Together, these data suggest that the inability of the Δ*crp*-*cya* mutant to metabolize carbon sources related to TCA cycle is likely a major reason for its attenuation.

*Salmonella* has a robust intracellular lifestyle, where it invades macrophages and resides inside a specialized compartment known as SCV. The SCV environment allows *Salmonella* to survive and replicate while being protected from macrophage attack. Compared to UK1, the Δ*crp*-*cya* mutant and its derivatives lacking *phoPQ, ompR-envZ, sifA* and *feoABC* exhibited severe growth defects and increased doubling times in an SCV-simulating conditions and were highly attenuated for invasion and intracellular survival within macrophages. All the four Δ*crp*-*cya* mutant derivatives were more attenuated than the mutant strain itself; of all the strains tested in this study, the derivatives lacking *ompR-envZ* and *feoABC* showed the highest attenuation. The *crp-cya* system represses the expression of SPI1, which is required for invasion (El Mouali et al., 2018). PhoPQ is a global regulator and regulates the expression of a number of downstream genes upon sensing the acidic environment of the SCV (Miller et al., 1989; Groisman, 2001). PhoPQ activates *grhD1*, which plays an important role in *S*. Typhimurium invasion (Banda et al., 2018). PhoPQ protects *S*. Typhimurium against reactive nitrogen species by regulating intracellular Mg^2+^ concentration (Bourret et al., 2017). PhoPQ-activated genes also protect *S*. Typhimurium from antimicrobial peptides produced by macrophages (Guo et al., 1998; Brodsky et al., 2002; Detweiler et al., 2003) and the acidic environment of the SCV (Bearson et al., 1998). The importance of OmpR-EnvZ in *S*. Typhimurium invasion and survival has previously been demonstrated in an *S*. Typhi infection model (Murret-Labarthe et al., 2020). OmpR-EnvZ activates the *ssrAB* two-component system which in turn induces the expression of SPI2 genes (Ochman et al., 1996; Cirillo et al., 1998; Hensel et al., 1998; Lee et al., 2000). SifA is an effector of T3SS-2 and plays a key role in maintaining the integrity of SCV and formation of *Salmonella*-induced tubules and is required for *Salmonella* virulence (Brumell et al., 2001; Knuff and Finlay, 2017). FeoABC is required for uptake of ferrous iron and helps *Salmonella* maintain iron homeostasis inside SCV (Kim et al., 2015; Wellawa et al., 2020). Given the key roles played by CRP-Cya, PhoPQ, OmpR-EnvZ, SifA and FeoABC in *Salmonella* invasion and/or intracellular survival, it is not surprising that the Δ*crp*-*cya* mutant and its derivatives are attenuated for invasion and/or intracellular survival in macrophages. The potential interaction of CRP-Cya with PhoPQ, OmpR-EnvZ, SifA and FeoABC likely explains the greater attenuation of the derivative mutants compared to the Δ*crp*-*cya* mutant.

Compared to UK1, the Δ*crp*-*cya* mutant derivatives lacking *ssrAB, SPI13, SPI2, sopF, sspH2, sseJ, mgtRBC* and *sitABCD* also exhibited severe growth defects in an SCV-simulating environment. However, compared to the Δ*crp*-*cya* strain, these derivatives showed increased invasion and intracellular survival within macrophages. SsrAB is a global transcriptional regulator activated soon after the internalization of *S*. Typhimurium by macrophages (Xu and Hensel, 2010). Activation of *ssrAB* leads to activation of *Salmonella* pathogenicity island (SPI) 2 and 13, which in turn activate expression of effectors such as SopF, SspH2, and SseJ (Cirillo et al., 1998; Xu and Hensel, 2010). Ion transporters such as MgtRBC and SitABCD help *Salmonella* maintain ion homeostasis inside SCV (Snavely et al., 1991; Kehres et al., 2002; Groisman et al., 2013). Despite the key roles played by SsrAB, SPI13, SPI2, SopF, SspH2, SseJ, MgtRBC and SitABCD in *S*. Typhimurium invasion and/or intracellular survival in macrophages, the increased invasion and intracellular phenotypes in the derivatives are likely the result of potential interaction of CRP-Cya with SsrAB, SPI13, SPI2, SopF, SspH2, SseJ, MgtRBC and SitABCD.

*Salmonella* infection is characterized by a marked global rearrangement of the macrophage transcriptome (Rosenberger et al., 2000; Saliba et al., 2016). Transcriptome profiling of macrophages infected with UK1, Δ*crp*-*cya* and its derivatives lacking *phoPQ, sifA* and *sopF* demonstrated that, compared to uninfected macrophages, 138, 148, 153, 155, and 142 genes were differentially expressed in these strains, respectively. Interestingly, despite being highly attenuated for invasion and intracellular survival in macrophages, the macrophage response to the Δ*crp*-*cya* mutant was similar to that of its parent strain UK1. Similarly, the derivatives lacking *phoPQ* and *sifA* were more attenuated in invasion and/or intra-macrophage survival than the Δ*crp*-*cya* mutant but the macrophage responses against these strains were similar to those of the Δ*crp*-*cya* mutant its parent strain.

**Figure 8:**
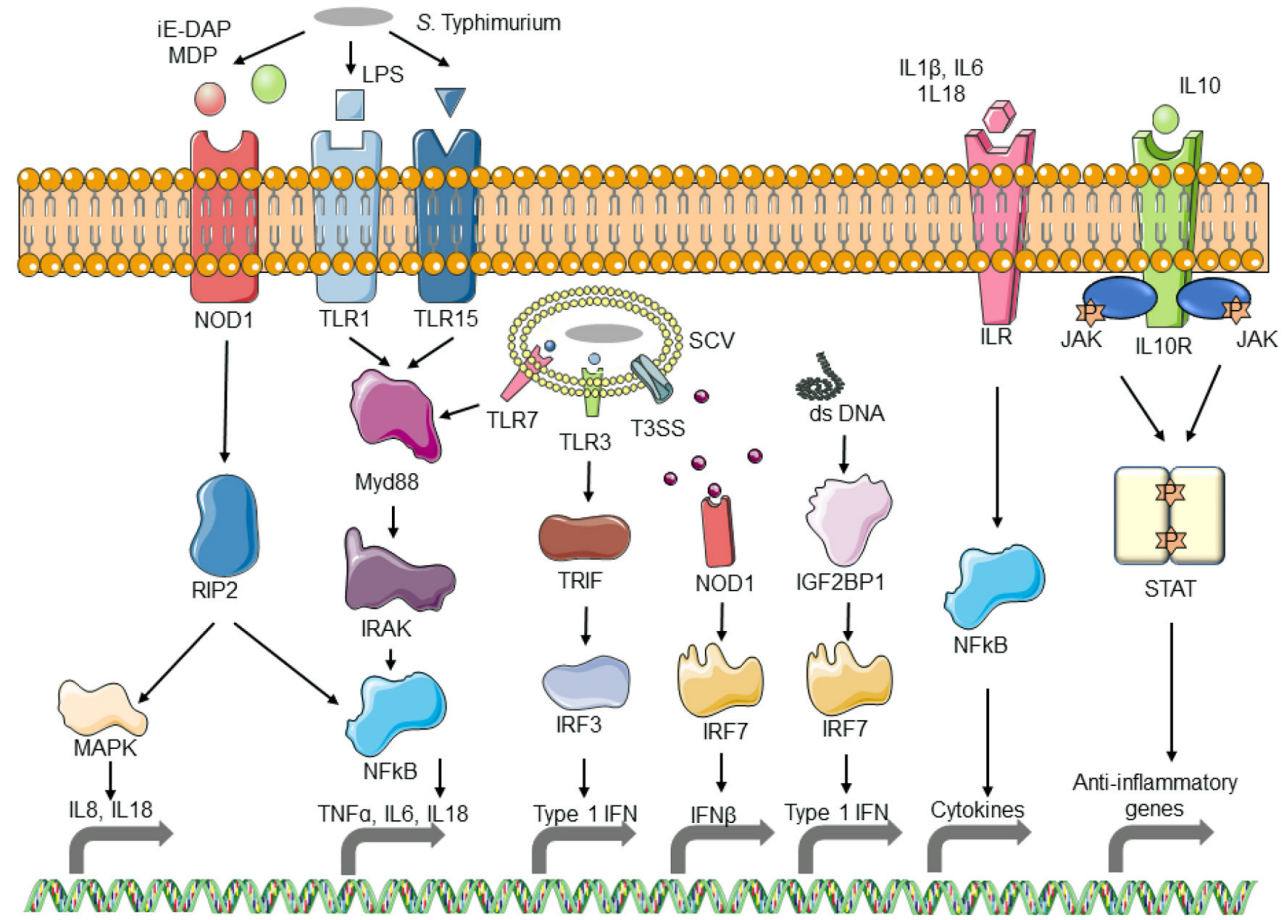
Schematic diagram of the response of macrophages to *Salmonella* infection. As *S*. Typhimurium comes in contact with macrophages, the pathogen-associated molecular patterns (PAMPs) are recognized by Toll-like receptors (TLRs) which activate the TLR pathway, resulting in the activation of interleukins and inflammatory cytokines. PAMPs from phagocytosed bacterial also activate TLR and Nod-like receptor (NLR) pathways. Bacterial DNA released into the macrophage cytosol is recognized by the cytosolic DNA sensing pathway and results in the activation of interferon. The resulting interleukins get recognized by interleukin receptors and initiate the expression of inflammatory cytokines.

Innate immune system plays a key role in counteracting pathogen attack by triggering immediate and early mechanisms of host defense and priming the adaptive immune system to produce antigen-specific antibodies. Infection of macrophages with UK1, the Δ*crp*-*cya* mutant and its derivatives upregulated several innate immune system genes involved in pathogen sensing and host defense. More specifically, genes belonging to Toll-like receptor signaling pathway, NOD-like receptor signaling pathway, cytosolic DNA sensing pathway, NF-κβ pathway, MAPK signaling pathway, and cytokine-cytokine receptor interaction were among the key upregulated genes. TLRs are upregulated in response to bacterial lipopolysaccharides (Yang et al., 1998; Chow et al., 1999). Activation of TLR signaling pathway cascades into the activation of NFκβ pathway. Activated NFκβ in turn induces the transcription of several genes that are involved in differentiation, inflammation, and cell survival (Baeuerle and Henkel, 1994; Medzhitov et al., 1997; Barkett and Gilmore, 1999). The NOD-like receptors (NLRs) also play an important role in pathogen recognition and signal induction of downstream genes. Unlike TLRs, NLRs are intracellular sensors that mainly recognize intracellular bacteria (Riordan et al., 2002). NLRs including both Nod1 and Nod2 recognize bacterial peptidoglycans and cascade the signal to induce the activation of NFκβ and MAPK pathway (Girardin et al., 2003; Hayden and Ghosh, 2004; McDonald et al., 2005). Signaling thorough NLRs results in a strong inflammatory response via secretion of proinflammatory cytokines (Travassos et al., 2005; Werts et al., 2007; Buchholz and Stephens, 2008); consistent with this, several genes encoding chemokines and cytokines were also upregulated in response to *Salmonella* infection. RIG-I is another cytosolic pathogen sensing system that senses short dsRNA and sRNA; infection with UK1, the Δ*crp*-*cya* mutant and its derivatives induced upregulation of genes in the RIG-I-like signaling pathway. Internalization of *Salmonella* also induces cell death by apoptosis, necroptosis, and autophagy (Gogoi et al., 2019). Consistent with these findings, several genes related to apoptosis and necroptosis were upregulated in macrophages infected with UK1, the Δ*crp*-*cya* mutant and its derivatives. Several genes were also downregulated in response to infection with *S*. Typhimurium strains. These genes belonged to cell cycle, carbohydrate metabolism, lipid metabolism, amino acid metabolism, ABC transporters, post-translational protein modification, and cellular senescence. This is supported by previous reports that host phagocytic cells reorganize their own metabolic pathways in response to bacterial internalization (Eisenreich et al., 2013; Olive and Sassetti, 2016). A schematic diagram showing the molecular mechanisms of macrophage response to *S*. Typhimurium infection is shown in Figure 8.

In summary, this study adds to our understanding of the mechanisms of virulence attenuation of the Δ*crp*-*cya* strain and provides novel insights on the early response of macrophages to this vaccine strain and its derivatives. Using primary chicken macrophages, our study also confirms the previously reported complex transcriptional response of macrophages to *Salmonella*. While showing higher attenuation for invasion and/or intracellular survival than the Δ*crp*-*cya* mutant, Δ*crp*-*cya*-Δ*phoPQ* and Δ*crp*-*cya*-Δ*sifA* mutants induced similar macrophage response to those of the Δ*crp*-*cya* mutant and its parent strain; it is worth exploring these strains as next-generation vaccine candidates with improved safety. Although we did not characterize the macrophage response of Δ*crp*-*cya*-Δ*ompR-envZ* and Δ*crp*-*cya*-Δ*feoABC*, of all the tested strains, these strains showed the highest attenuation for invasion and intracellular survival in macrophages; it is also worth exploring these strains as additional vaccine candidates to reduce *Salmonella* burden in poultry.

## Data Availability Statement

The datasets presented in this study can be found in online repositories. The name of the repository and accession number can be found below: GEO, NCBI: GSE193063.

## Author Contributions

AK, DG, and BB conceived and designed the experiments. BB performed the experiments. BB and DG analyzed and interpreted the data and wrote the manuscript. All authors read, reviewed, and approved the final manuscript.

## Conflict of Interest

BB, AK, and DG are employees of Elanco Animal Health, Inc. Elanco Animal Health, Inc. is a company that develops, manufactures and sells veterinary pharmaceuticals and nutritionals.

## Publisher's Note

All claims expressed in this article are solely those of the authors and do not necessarily represent those of their affiliated organizations, or those of the publisher, the editors and the reviewers. Any product that may be evaluated in this article, or claim that may be made by its manufacturer, is not guaranteed or endorsed by the publisher.
